# High-resolution DIC analysis of in situ strain and crack propagation in coated AZ31 magnesium alloys under mechanical loading

**DOI:** 10.1007/s10853-025-11243-4

**Published:** 2025-08-25

**Authors:** Berzah Yavuzyegit, Katerina Karali, Sarah Davis, Benjamin Morrison, Suleyman Karabal, Kemal Balandiz, Nigel Smith, Sergey Usov, Pavel Shashkov, Roxane Bonithon, Gordon Blunn

**Affiliations:** 1 Faculty of Science and Health, School of Medicine, Pharmacy and Biomedical Sciences, University of Portsmouth, St Michael’s Building, White Swan Road, Portsmouth, PO1 2DT UK; 2 Department of Mechanical Engineering, Recep Tayyip Erdogan University, 53100 Rize, Türkiye; 3 School of Mechanical & Design Engineering Faculty of Technology, University of Portsmouth, Anglesea Building, Anglesea Road, Portsmouth, PO1 3DJ UK; 4 Department of Mechanical Engineering, Abdullah Gül University, 38080 Kayseri, Turkey; 5 Alten UK Innovation Laboratory, Alten Limited, 8 Pinnacle Way, Pride Park, Derby, DE24 8ZS UK; 6 BioCera Medical Limited, 3B Homefield Road, Haverhill, Suffolk CB9 8QP UK

## Abstract

Biodegradable magnesium (Mg) alloys are promising for various biomedical applications but their susceptibility to corrosion poses significant challenges. This study systematically examines the microstructural integrity and failure mechanisms of electrochemically deposited phosphate- and fluorine-rich coatings on AZ31 Mg alloy subjected to three-point bending (3 PB) in both non-corrosive and physiological (HBSS) environments. High-resolution digital image correlation (HR-DIC) combined with scanning electron microscopy (SEM) enables in situ visualization and quantitative analysis of crack initiation, evolution, and propagation within the coatings. Our findings reveal that thinner (5 µm) coatings are prone to forming dense networks of fine cracks, while thicker (15 µm) coatings display fewer but wider cracks, with both morphologies strongly governed by localized shear strain. Importantly, cross-sectional analyses after load-holding demonstrate that, while surface cracks initially remain confined within the coating, cracks generated under higher mechanical loading can propagate through the entire coating thickness. These through-thickness cracks create direct pathways for corrosive fluids to access the underlying alloy, serving as initiation sites for stress corrosion cracking within the substrate. Furthermore, our results indicate that fluoride in the coating mitigates rapid corrosion. Overall, the study reveals that coating failure and the formation of through-thickness cracks play a critical role in facilitating localized corrosion and crack initiation within the alloy under combined mechanical and corrosive environments.

**Graphical Abstract:**

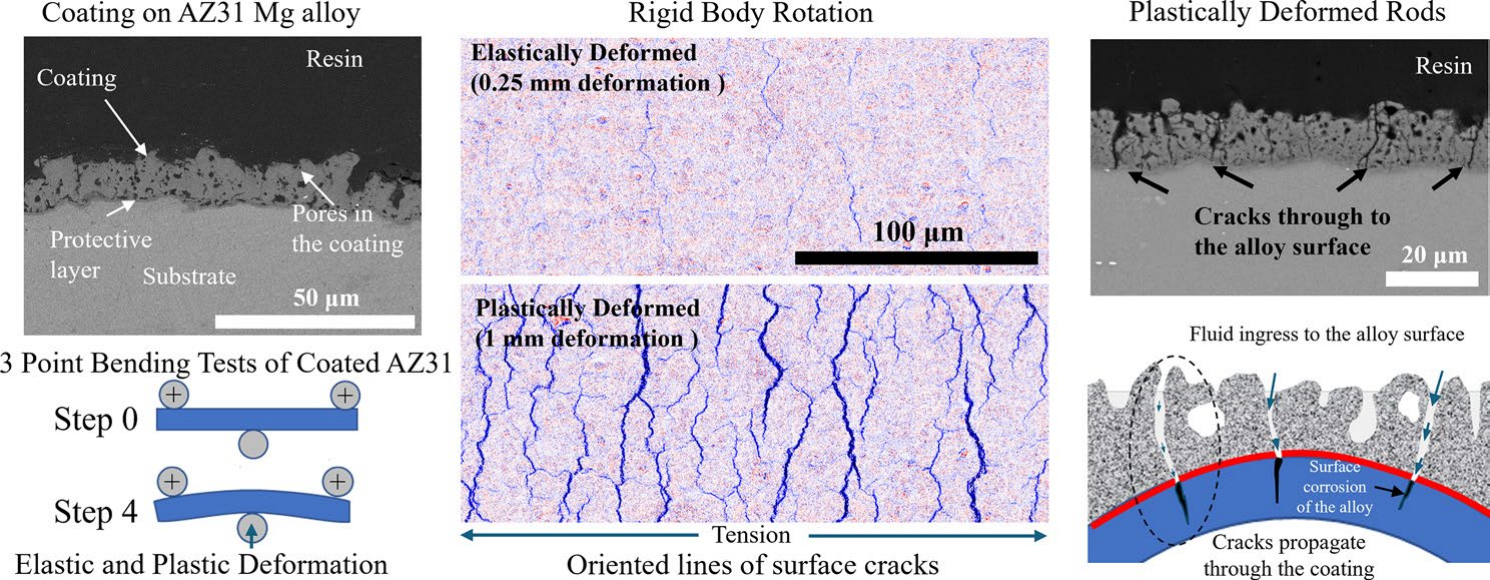

**Supplementary Information:**

The online version contains supplementary material available at 10.1007/s10853-025-11243-4.

## Introduction

Mg and Mg alloys have attracted considerable attention as potential biomedical materials for orthopaedic devices such as bone screws and fracture plates, as well as for biodegradable vascular interventions such as stents [1–3]. These implants are designed to naturally degrade, thereby eliminating the need for a secondary surgical procedure to remove the device [4, 5]. For orthopaedic applications, the strength and density of these materials closely match those of cortical bone [6–8] and Mg-based implants have the potential to stimulate bone cell proliferation [9], accelerate the bone healing process, and avert stress shielding effects due to their elastic modulus which is similar to bone than other metallic alloys used [4]. A resorbable implant must maintain sufficient strength for its intended use under load while also having the ability to degrade in a controlled manner [10, 11]. However, in chloride-rich saline environments found in the body, the implants' susceptibility to corrosion is increased, risking structural integrity and performance [12–14]. Their corrosion leads to the evolution and accumulation of hydrogen gas [15], which can undermine tissue integration [16, 17] and induce swelling in tissues [18]. To fully utilize Mg alloys' potential in biomedical applications, it is imperative to improve their corrosion resistance, and one of the ways that this is achieved is through coatings [19]. Modifying the surface of Mg alloys using surface treatments or coatings can increase their corrosion resistance, enhancing their biological characteristics such as osteointegration and osteoconduction [20, 21] and their mechanical properties [22].

Among various coatings, plasma electrolytic oxidation (PEO) coating offers robust corrosion resistance and mitigates hydrogen release from these alloys [15, 23]. The technique can produce coatings of different thicknesses that are strongly adherent to the substrate with relatively low porosity, enabling tailored protection and controlled corrosion. In a previous study [24], we assessed the effect of electrochemically oxidized (ECO) coatings on the corrosion resistance of AZ31 alloy. ECO coating is a patented form of the PEO process. The results from this previous study showed that the coatings enhanced the corrosion resistance of the alloy, highlighting their potential for corrosion mitigation in biomedical applications. However, this study was carried out on non-loaded samples, and orthopaedic implants experience static and dynamic loads resulting from activities such as walking and running [25]. The strain induced by these activities may affect coating adhesion [20] and subsequent corrosion.

Recent studies have underscored the importance of understanding how mechanical deformation influences the integrity and corrosion performance of protective coatings [26]. In industrial systems, in situ investigations [27, 28] utilized advanced techniques like SEM-based mechanical testing and high-resolution digital image correlation (HR-DIC) to monitor strain localization and crack evolution in coatings such as Zn–Al–Mg and TiAlN. These studies revealed that brittle phases [29], heterogeneous deformation, and strain concentration can significantly promote crack initiation and propagation, while certain oxide layers (e.g. Al_2_O_3_ (alumina)) may still offer residual corrosion protection [30]. However, despite these advancements, the current literature largely focuses on rigid or industrial coatings under simplified loading conditions. There remains a critical lack of high-resolution, in situ analyses specifically targeting PEO-coated biodegradable magnesium alloys where these materials are being increasingly considered for load-bearing resorbable implants. Given the physiological and mechanical stresses encountered in clinical applications, such testing is essential to evaluate coating integrity.

Mg shows heterogeneous strain distribution on the nanoscale during deformation [31] while on the surface, the coating separates into smaller segments, leading to the redistribution and relaxation of localized stress [32, 33]. This is particularly important for coatings that have different material properties from the underlying substrate, as the relative strain differential will lead to high shear stress at the coating interface that may lead to cracking and delamination of the coating, resulting in the onset of corrosion. However, the strain characteristics and stress transfer mechanisms between interfaces during the elastic and plastic deformation of coatings are rarely reported [34, 35]. Additionally, the small size of features such as pores and cracks in PEO coatings complicates strain analysis via conventional methods. In this study, we employed scanning electron microscopy (SEM) combined with high-resolution, non-contact, and non-destructive Digital Image Correlation (HR-DIC) to examine how strain affects coating integrity and if this increases the corrosion of the underlying alloy.

## Materials and methods

### Sample preparation

AZ31 Mg alloy (Mg–Al 3 wt%, Zn 1wt%) rods 3 mm in diameter were cut to a length of 115 mm (Goodfellow Cambridge Limited-UK, Huntingdon, UK). The soft-sparking PEO used in the applied surfacing technique was created and patented by Biocera Medical Ltd. (PCT publication WO 2020049299), which is described elsewhere [24]. The goal of this method, known as electrochemical oxidation (ECO), is to reduce sparking associated with conventional PEO coatings. With surface roughness regulated by the electrical parameters, ECO produces a denser and more compact layer by applying positive and negative electrical pulses. The process alternates between galvanostatic and potentiostatic modes to encourage the development of a regulated nanocrystalline ceramic structure and avoids breakdown discharge sparking.

Water-based solutions with fluoride and phosphates at concentrations usually less than 5 g/L make up the electrolyte. The potassium hydroxide (KOH) concentration is adjusted to preserve the electrolytes' electrical conductivity at 25 mS [18]. Fluoride compacts the coating and may improve its antibacterial qualities, whilst phosphate in the coating maintains the nanoceramic layer's hardness and biocompatibility [36]. Two coating thicknesses were used, one measuring 5 µm thick and the other measuring 15 µm thick.

The in situ HR-DIC 3 PB test specimens were cut to Ø3 mm × 35 mm in length. The end surfaces were ground with 800 grit silicon carbide paper. After 15 min of ultrasonic cleaning (XUBA3, Grant Instruments, Cambridgeshire, UK) in 99% + ethanol, each sample was allowed to air dry for 30 min.

### Corrosion tests

Two uncoated and two 15 μm ECO-coated magnesium samples were submerged in 100 mL of Hank's Balanced Salt Solution (HBSS) (Gibco, Thermo Fisher, USA) for two weeks on an orbital shaker (Orbi-Shaker, Benchmark, USA) at a set speed of 30 rpm. The HBSS solution comprised 0.35 g/L NaHCO_3_, 1 g/L glucose, 8 g/L NaCl, 0.4 g/L KCl, 0.04 g/L Na_2_HPO_4_, 0.06 g/L KH_2_PO_4_, 0.20 g/L MgSO_4_· 7H_2_O, and 0.14 g/L CaCl_2_ (Gibco, Thermo Fisher, USA). Weight loss was used to calculate the corrosion rate of the samples.

After the samples were removed, ultrasonically cleaned in ethanol for 15 min to eliminate corrosion products, allowed to air dry for 30 min, weighed, and then suspended again in the same HBSS solution. Weight readings were made every two days. The following formula was used to determine the corrosion rate:

1 $$\text{Corrosion Rate}=\frac{{W}_{\text{loss}}}{\rho \times \text{A}\times t}$$

where ${W}_{\text{loss}}$ represents the weight loss, $\rho$ is the density (1.738 g/cm^3^), *A* denotes the total area of the rods undergoing corrosion, and the exposure time in the corrosive environment is denoted by *t*.

### Three point bending tests of AZ31 Mg alloy

To evaluate the flexural properties of the rod samples, a force–displacement curve was made using 3 PB conducted on uncoated and coated non-corroded AZ31 Mg alloy samples. 3 PB tests were conducted using a Bose Electroforce 3000 (Massachusetts, USA) using a support span of 30 mm and acetal support rollers with a diameter of 3 mm, replicating the setup used in the HR-DIC stage. A constant loading rate of 1 N/s was applied until the machine’s maximum force capacity of 150 N was reached.

For each group, three coated samples were tested (*n* = 3), in addition to three uncoated samples. The bending stress (*σ*) and strain (*ε*) were calculated using standard 3 PB equations:

2 $$\sigma = \frac{FL}{{\pi R^{3} }}\;{\text{and}}\;\varepsilon = \frac{6Dd}{{L^{2} }}$$

where *F* is the applied load, *L* is the support span (30 mm), *d* is the specimen diameter, *R* is the specimen radius, and *D* is the mid-span deflection. All geometrical parameters were measured with a digital caliper prior to testing. These values were used to generate flexural stress–strain curves for comparative evaluation.

### In situ loading and SEM imaging

**Figure 1:**
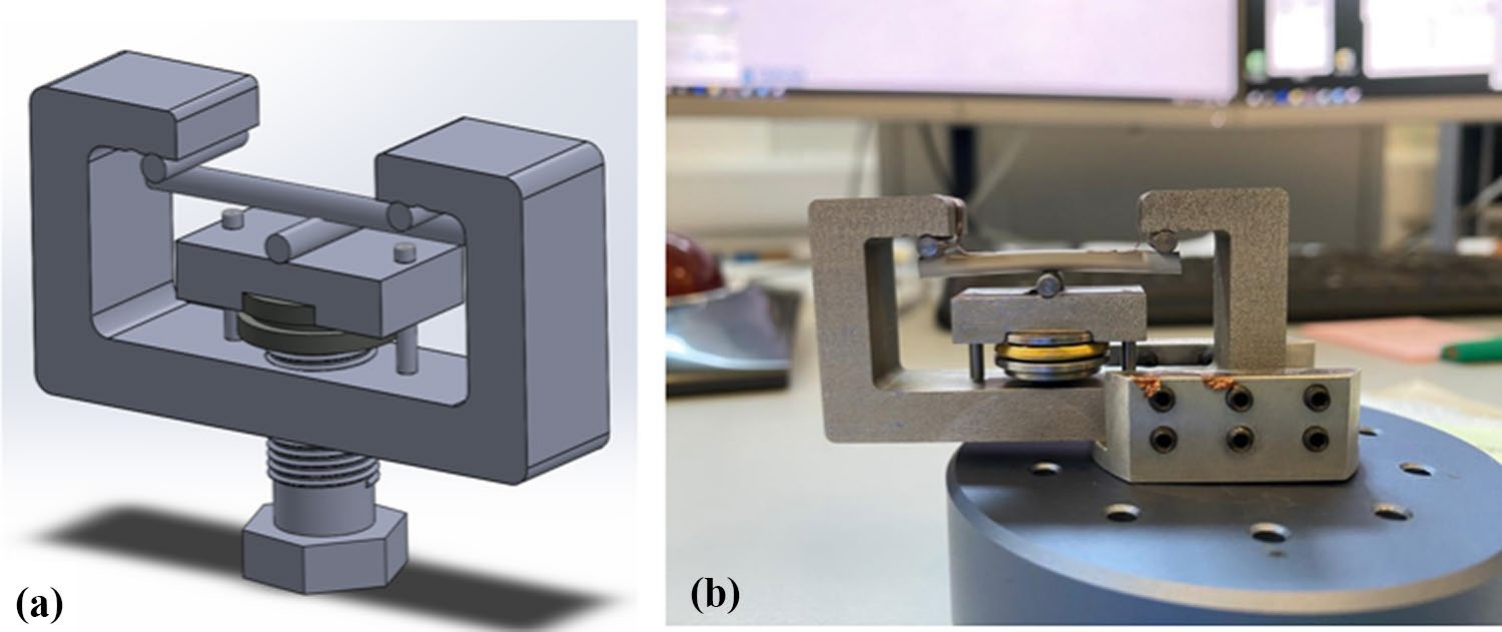
**a** Schematic representation of the static loading in situ SEM test rig, and **b** the static loading in situ SEM test rig in operation

The in situ 3 PB experiments were carried out at room temperature, with the focal point of the SEM images being moved incrementally by up to 1 mm. This was accomplished with a specially designed in situ 3 PB testing apparatus that had pin sizes of 3 mm in diameter and a supporting length of 30 mm. The pins were made of acetal and were designed to prevent galvanic corrosion while ensuring an even distribution of localized stress at the contact points with the rod (Fig. 1). Only the tension surface was investigated.

The load was applied to the samples by an M12 screw with a 1 mm pitch size, translating one rotation into 1 mm displacement. Furthermore, the screw head was tailored to accommodate an allen key, ensuring gradual and controlled application of load. The compact height of the rig allowed it to be placed within the TESCAN Mira3 FEG-SEM OI (Brno-Kohoutovice, Czech Republic) chamber. To ensure optimal vertical load transmission, a thrust-bearing mechanism was incorporated into the apparatus.

For in situ imaging, secondary electron (SE) images were captured at a working distance of 16 mm, using a dwell time of 3 microseconds per pixel, an acceleration voltage of 5 kV, and a beam current of 1 nanoampere. Images were captured at 500× to 10000× magnification, depending on crack visibility. Displacement-controlled loading was employed with each increment of load applied by a quarter of a screw rotation (corresponding to 0.25 mm displacement) which was accurately measured by the working distance of the focused image in the SEM. During each stage, a grid of 12 × 12 SE images with 10% overlap was captured and stitched together, encompassing a total area of 300 × 225 µm^2^ that was used for the analysis. With a field of vision of 30 µm and a 10% overlap between neighbouring images, each individual image, or tile, was made up of 2048 pixels by 2048 pixels. SE imaging was performed once at stages of 0.25 mm (step 1), 0.5 mm (step 2), 0.75 mm (step 3), and 1 mm (step 4), as well as twice prior to loading (step 0).

### In situ strain mapping by HR-DIC

The HR-DIC analysis was performed by comparing the stitched high-resolution SEM images acquired during in situ 3 PB testing with the original undeformed condition using the LaVision program DaVis® (version 8.4.0) (LaVision GmbH, Germany). A subset size of 12 × 12 pixels (175 × 175 nm^2^) was chosen to balance strain resolution and spatial resolution. Strain resolution study revealed that the minimal error rate was ± 0.1 pixels (17.5 nm). With the tensile axis parallel to the x_1_ direction, in-plane displacement fields were created as u (*x*_1_, *x*_2_, 0), where *x*_1_ corresponds to the sample axis (rolling direction, RD), and *x*_2_ denotes the vertical or transverse direction (TD), i.e. the loading direction during three-point bending. Local in-plane deformation was calculated by differentiating the displacement fields at the centre of each subset, yielding the following strain tensor:

3 $${\varepsilon }_{ij}=\left[\begin{array}{cc}\frac{\text{d}u}{{\text{d}x}_{1}}& \frac{\text{d}v}{{\text{d}x}_{1}}\\ \frac{\text{d}u}{{\text{d}x}_{2}}& \frac{\text{d}v}{{\text{d}x}_{2}}\end{array}\right]$$

To characterize localized deformation and crack-prone zones, effective shear strain (${\gamma }_{eff}$) maps were computed using the following relation:

4 $${\gamma }_{\text{eff}}=\sqrt{{\left(\frac{{\varepsilon }_{11}-{\varepsilon }_{22}}{2}\right)}^{2}+{\varepsilon }_{12}^{2}}$$

here ${\varepsilon }_{11}=\frac{\text{d}u}{{\text{d}x}_{1}}$ represents the normal strain along the sample axis (longitudinal direction), ${\varepsilon }_{22}=\frac{dv}{{dx}_{2}}$ is the normal strain along the loading axis (vertical direction), and ${\varepsilon }_{12}=\frac{1}{2}\left(\frac{\partial u}{{\partial x}_{2}}+\frac{\partial v}{{\partial x}_{1}}\right)$ is the in-plane shear strain component. Unless otherwise noted, the stresses computed for each stage indicate the total cumulative strain. The following formula was used to calculate the rigid body rotations (RBR) orthogonal to the sample surface (RD-TD): $\text{RBR}= \frac{1}{2}\left(\frac{\text{d}u}{{\text{d}x}_{2}}-\frac{\text{d}v}{{\text{d}x}_{1}}\right)$.

RBR maps were utilized for crack analysis of the DIC maps because they effectively highlighted discontinuities associated with crack formation and propagation. Unlike tensile strain or ${\gamma }_{\text{eff}}$ maps, which may include noise and less distinct features, RBR maps provide a clearer visualization of crack behaviour. RBR refers to the rotational movement of a material or structure without deformation, indicating areas where material segments move as rigid bodies, which is typically seen around crack regions. This clarity in visualizing cracks allows for more accurate identification and tracking of active cracks during the testing process.

By employing RBR maps, we ensured a more precise and reliable identification of crack locations and behaviour, allowing for the precise delineation of segmented lines along these active cracks for detailed analysis.

#### Crack orientation angle analysis

High-resolution SEM images were captured at both the initial (step 0, undeformed) and final deformation stages (step 4). Crack paths were manually traced using ImageJ software, segmenting each crack into distinct linear segments to accurately measure orientation angles [37]. These angles, defined relative to the sample’s longitudinal axis, ranged from 0° (parallel to the tensile axis) to 90° (perpendicular to the tensile axis). The measured angle data were compiled into histograms to visualize the distribution of crack orientations. This analysis was performed separately for each sample type: uncorroded 5 µm coating, uncorroded 15 µm coating, and corroded 15 µm coating, at both step 0 and step 4.

#### Crack displacement quantification

**Figure 2:**
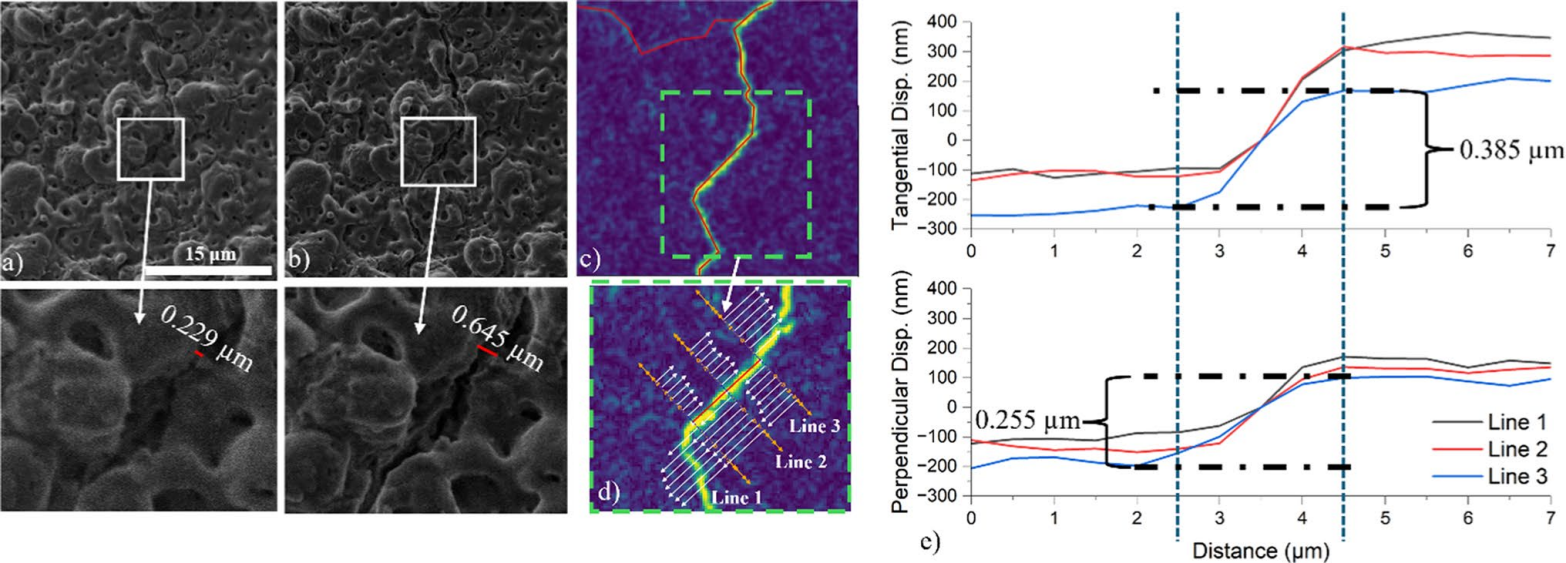
Analysis of crack initiation and propagation using HR-DIC data and Python. **a** and **b**: SEM images showing crack regions before and after testing, respectively. The inlet images show the crack thickness before and after the deformation. **c** ${\gamma }_{\text{eff}}$ maps with crack lines (red) defined using ImageJ. **d** Insets show orthogonal lines (10 pixels each side) to the crack line used for displacement analysis. Displacement vectors along these lines were used to analyse crack displacement. **e** Tangential and perpendicular displacement profiles along the crack line (Lines 1, 2, and 3)

HR-DIC was used to compute displacement and strain fields from stitched SEM image grids (Fig. 2a–b) at each deformation step. The initial displacement gradient computations were performed using the open-source Python package DefDAP [38], which is tailored for digital image post-processing in high-resolution deformation studies. These steps allowed for the generation of ${\gamma }_{\text{eff}}$ and RBR maps (Fig. 2c). Following strain field computation, the crack displacement analysis consisted of the following key steps:

1. **Crack Line Definition** Cracks were manually segmented using ImageJ based on ${\gamma }_{\text{eff}}$ and RBR maps. These cracks were defined by short, straight-line segments, and the start and end coordinates were extracted for each crack line (Fig. 2c).
2. **Orthogonal Displacement Sampling** A custom methodology originally developed in Yavuzyegit's doctoral thesis [39] was used to compute localized crack-opening behaviour. Orthogonal lines (± 10 pixels each side) were drawn across each crack segment midpoint to capture displacements on either side of the crack path (Fig. 2c inlet-Line 1). The displacement vectors for each pixel along the orthogonal line were determined by comparing the displacement values from (*x*_start_, *y*_start_) to (*x*_stop_, y_stop_). To account for local deformation and minimize global deformation effects, each line's vectors are subtracted from the local displacement values (*x*_*i*_, *y*_*i*_) at the chosen region's midpoint. These lines were used to analyse displacement both along and perpendicular to the crack.
3. **Projection of Displacement Vectors** The tangential (*t*_*x*_, *t*_*y*_) and perpendicular (*p*_*x*_, *p*_*y*_) displacements were calculated at multiple points by projecting the displacement vectors onto the unit vectors of the orthogonal lines (Fig. 2e).
4. **Calculation of Crack Widening** The extent of the crack widening was calculated by subtracting the projected values 1 µm away from the midpoint on each side (shown in Fig. 2e by blue dashed lines), where significant offsets occur, for the tangential (${\delta }_{\text{t}}$) and perpendicular (${\delta }_{\text{p}}$) displacements. Then, the magnitude of the crack widening was calculated using the formula:
  5 $$\delta =\sqrt{{\delta }_{\text{t}}^{2}+{\delta }_{\text{p}}^{2}}$$
5. **Averaging Across Crack Length** The mean widening of a crack was calculated by averaging individual segment openings along its length using:
  6 $${\delta }_{\text{avg}}=\frac{1}{n}{\sum }_{i=1}^{n}\sqrt{{({\delta }_{{\text{t}}_{i}})}^{2}+{({\delta }_{{\text{p}}_{i}})}^{2}}$$
  where n is the number of point pairs, ${\delta }_{{\text{t}}_{i}}$ and ${\delta }_{{\text{p}}_{i}}$ represent the tangential and perpendicular displacements for the pair of points, respectively.
6. **Validation** Quantitative results from HR-DIC were validated by comparing to SEM images before and after loading. Inset images and crack overlay maps confirmed that DIC-based displacement measurements correspond to the observed physical widening of cracks.

### Microstructural characterization after in situ 3BP

Following the in situ 3 PB tests, cross sections of the samples and the coatings were examined. To capture the effect of the loads on the coatings, samples were embedded under 3 PB. The cross-sectioning encompassed regions that included both longitudinal and transverse directions, allowing for investigation of both compression and tension sides of the samples. The rods were embedded in resin (Metprep, UK) and underwent sectioning, then grinding with silicon carbide papers of 800, 1200, and 4000 grit, then polishing using oil-based diamond suspension (1 and 0.25 μm) (MetPrep, UK). The samples were cleaned with ethanol in an ultrasonic bath for fifteen minutes.

TESCAN Mira3 (FEG-SEM) (Czech Republic) was used to examine the cross-sectional morphology and energy-dispersive X-ray spectroscopy (EDX) spectra of the samples. Representative regions were selected for analysis to investigate the composition and structure. Images of SE and BSE were obtained using a working distance of 12 mm, a beam current of 0.8 nanoamperes, and an acceleration voltage of 5 kV. EDX images were acquired using a beam current of 1 μA, an acceleration voltage of 15 kV, and a working distance of 15 mm.

### Static loading of coated and uncoated AZ31 Mg alloy

Static loading tests were conducted in 100 ml of HBSS solution over 15 days. A custom-made 3 PB test rig with a 30 mm support span and 3 mm pin diameters was employed, which allowed for controlled displacement increments. To mitigate galvanic corrosion, the pins, made of acetal, ensuring uniform stress distribution at contact points with the rod. A loading pin, positioned on a notched acetal piece aligned by longer acetal pins, applied the load.

The load was applied via displacement control using an M14 screw, with load measurement facilitated by a load cell beneath the setup. Initially, a static load of 1471 g, equivalent to 150 N, was applied at room temperature using 15 µm coated and uncoated Mg alloy samples (*n* = 3). Due to the compliance in the test rig, load relaxation occurred, so the load was increased to 150 N every half hour for the first hour. Changes in load were measured throughout the test. After the test, the surface and cross sections of the rods were investigated using SEM.

## Results

### Morphology of coating

**Figure 3:**
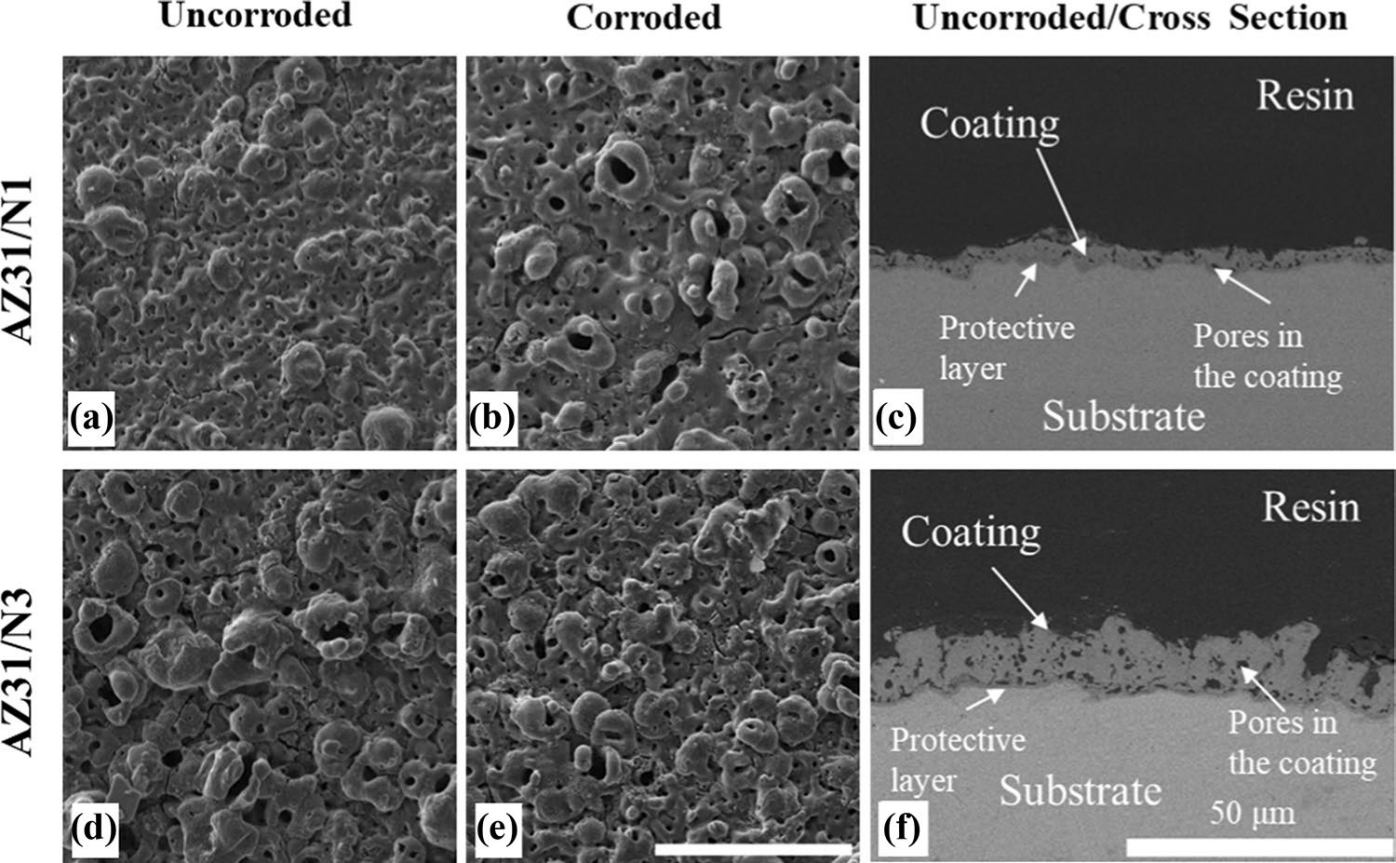
Surface morphology and cross-sectional views of uncorroded and corroded samples. **a**–**c** 5 µm sample: **a** surface morphology before immersion, **b** after corrosion, and **c** cross-sectional view. **d**–**f** 15 µm sample: **d** surface morphology before immersion, **e** after corrosion, and **f** cross-sectional view

To investigate the effects of loading on the integrity of the coating and the corrosion behaviour of the coated alloys, the surface and cross-sectional morphology of the ECO-coated AZ31 Mg alloy was examined using SEM (Fig. 3). The secondary electron SEM images revealed distinct differences in surface features between the 5 µm and 15 µm coatings. The 5 µm coating displayed a relatively smooth and more uniform surface, while the 15 µm coating exhibited a rougher, more uneven texture. The uncorroded samples predominantly featured small, circular pores, whereas the corroded samples showed larger, circular pores.

A comparative analysis was conducted to evaluate the differences in corrosion behaviour between the coated samples. The corrosion rate of the uncoated and 15 µm coated AZ31 Mg samples in HBSS at room temperature is determined by Eq. 1. Following a 14-day immersion in HBSS, no noticeable weight loss was observed in uncoated and 15 µm coated rods, indicating a low corrosion rate of 10^−5^ ± 10^−5^ mm/year.

Energy-dispersive X-ray spectroscopy (EDX) was used to assess the elemental composition of the uncorroded coatings. As detailed in our previous study [24], the coatings exhibit a layered structure consisting of a dense fluoride-rich barrier (~ 1 µm) adjacent to the AZ31 substrate, and a microporous outer layer enriched with magnesium, phosphorus, and fluoride. In the present study, cross-sectional EDX analysis confirmed this morphology and yielded average coating thicknesses of 3.39 ± 0.8 µm and 11.5 ± 1.7 µm for the nominal 5 µm and 15 µm coatings, respectively. Surface-connected pores and cracks were observed to extend into the coating without breaching the fluorine-rich interface.

### Mechanical properties of AZ31 in 3-point bending

**Figure 4:**
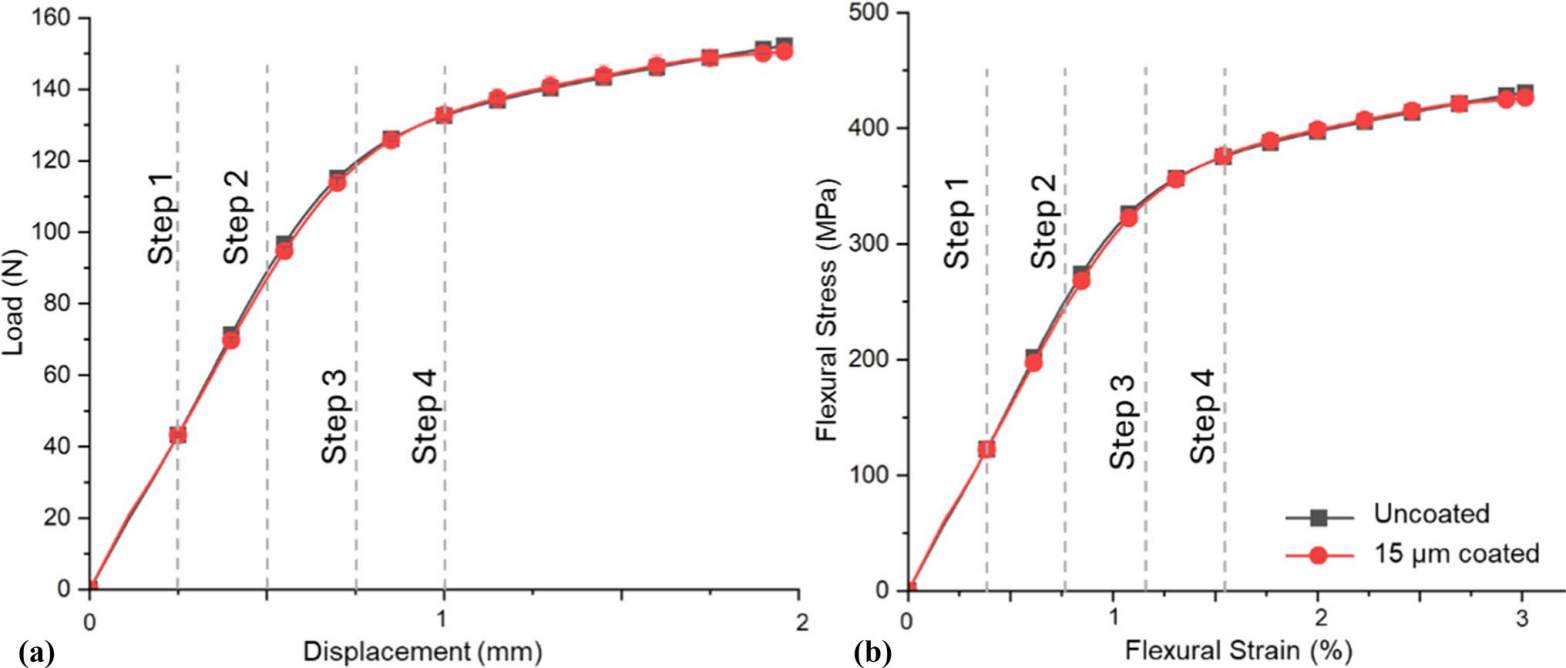
**a** Load–displacement and **b** Flexural stress–strain curves from 3 PB tests of uncoated and 15 µm coated AZ31 Mg alloy

To investigate the effects of loading on the coating, the flexural properties of the rods were investigated using load–displacement characteristics of the uncoated AZ31 Mg alloy rods (Fig. 4) through continuous 3 PB tests. The alloy's average elastic modulus and yield strength were determined to be 25 ± 5 GPa and 290 ± 10 MPa, respectively. The samples did not reach either the ultimate strength or the failure strength due to limitations in the maximum load capacity of the equipment used (150 N). Displacements of 0.25 mm (step 1) and 0.5 mm (step 2) were within the elastic region, while displacements of 0.75 mm (step 3) and 1 mm (step 4) were in the plastic region. These displacements were subsequently used in the HR-DIC tests to investigate the coating integrity under 3 PB.

### In situ 3 PB testing of the coated AZ31 Mg alloy

To investigate the mechanical integrity of phosphate-based coatings on AZ31 magnesium alloy under mechanical stress, in situ three-point bending (3 PB) tests were conducted within a scanning electron microscope (SEM). Samples with coating thicknesses of 5 µm and 15 µm, both corroded and uncorroded, were incrementally deformed up to 1 mm (step 4) displacement. The deformation process was systematically monitored to assess crack initiation, propagation, and orientation using high-resolution digital image correlation (HR-DIC) combined with SEM imaging.

#### Crack initiation

**Figure 5:**
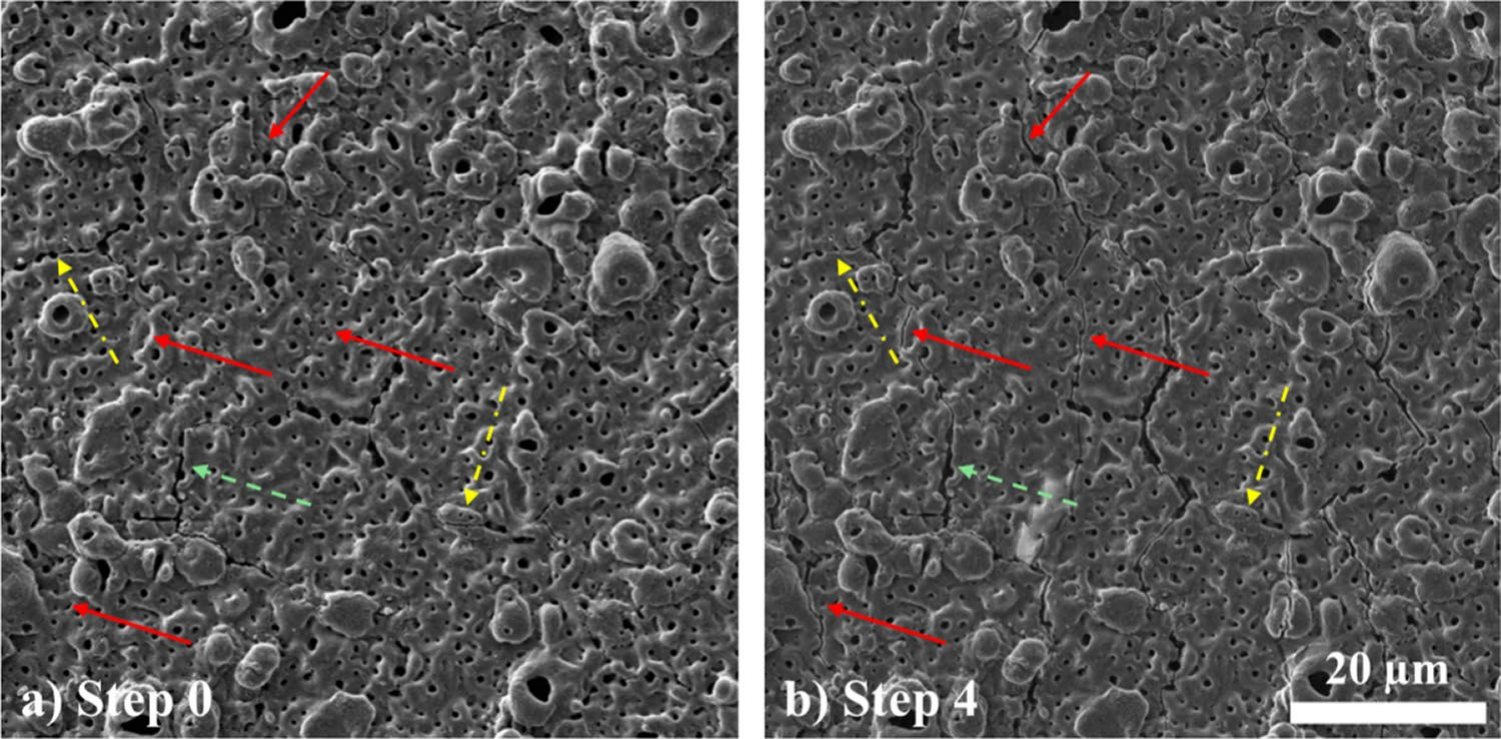
High-magnification SEM images of the same region on the surface of an uncorroded 5 μm coated AZ31 Mg alloy sample: **a** initial state (step 0), and **b** after deformation with 1 mm displacement (step 4). Red arrows indicate newly formed cracks that are not present at step 0 but appear after mechanical loading. Yellow dashed-dot arrows mark pre-existing cracks that show little or no change (non-active cracks) between step 0 and step 4. Green dashed arrows highlight cracks that are present at step 0 and open further under loading (active cracks)

To facilitate clearer interpretation of crack evolution behaviour, Fig. 5 presents high-magnification SEM images of the same region on the uncorroded 5 μm coated AZ31 Mg alloy surface before and after mechanical loading. Distinct crack types are visually differentiated: newly formed cracks (red arrows) appear only after loading, non-active cracks (yellow dashed-dot arrows) exist prior to loading but show little or no change in width, and active cracks (green dashed arrows) widen significantly under load.

#### Crack orientation and network evolution

The initial (as-received, step 0) and final crack orientations (at step 4) for both the uncorroded 5 μm and 15 μm coated samples, as well as the corroded 15 μm coated Mg alloys, were tracked by manually defining crack lines on stitched SEM images obtained during the in situ 3 PB tests (Figure S1). More than 2500-line segments were analysed for each image. For the 5 μm coating, in the initial state (step 0), most of the cracks merge to form a closed network, whereas network formation was less obvious for the 15 μm coating in the initial (step 0) images. In the deformed state (step 4), the 5 μm coating creates a network of cracks, whereas small-segmented lines are observed on the 15 μm coatings. Compared to the cracks formed after the deformation, the initial cracks are generally curved, while the new cracks are more linear for all the samples.

Normalized crack lengths, calculated by dividing the total crack length by the area observed, increased significantly with deformation. For the uncorroded samples with 5 µm coating thickness, the normalized crack length increased from 0.0695 ± 0.01 µm/µm^2^ at step 0 to 0.1025 ± 0.016 µm/µm^2^ at step 4, indicating a 47.5% increase in crack length. Similarly, the uncorroded sample with a 15 µm coating showed a 45.1% increase in normalized crack length. In contrast, the corroded sample with a 15 µm coating exhibited a 56.12% increase in normalized crack length.

**Figure 6:**
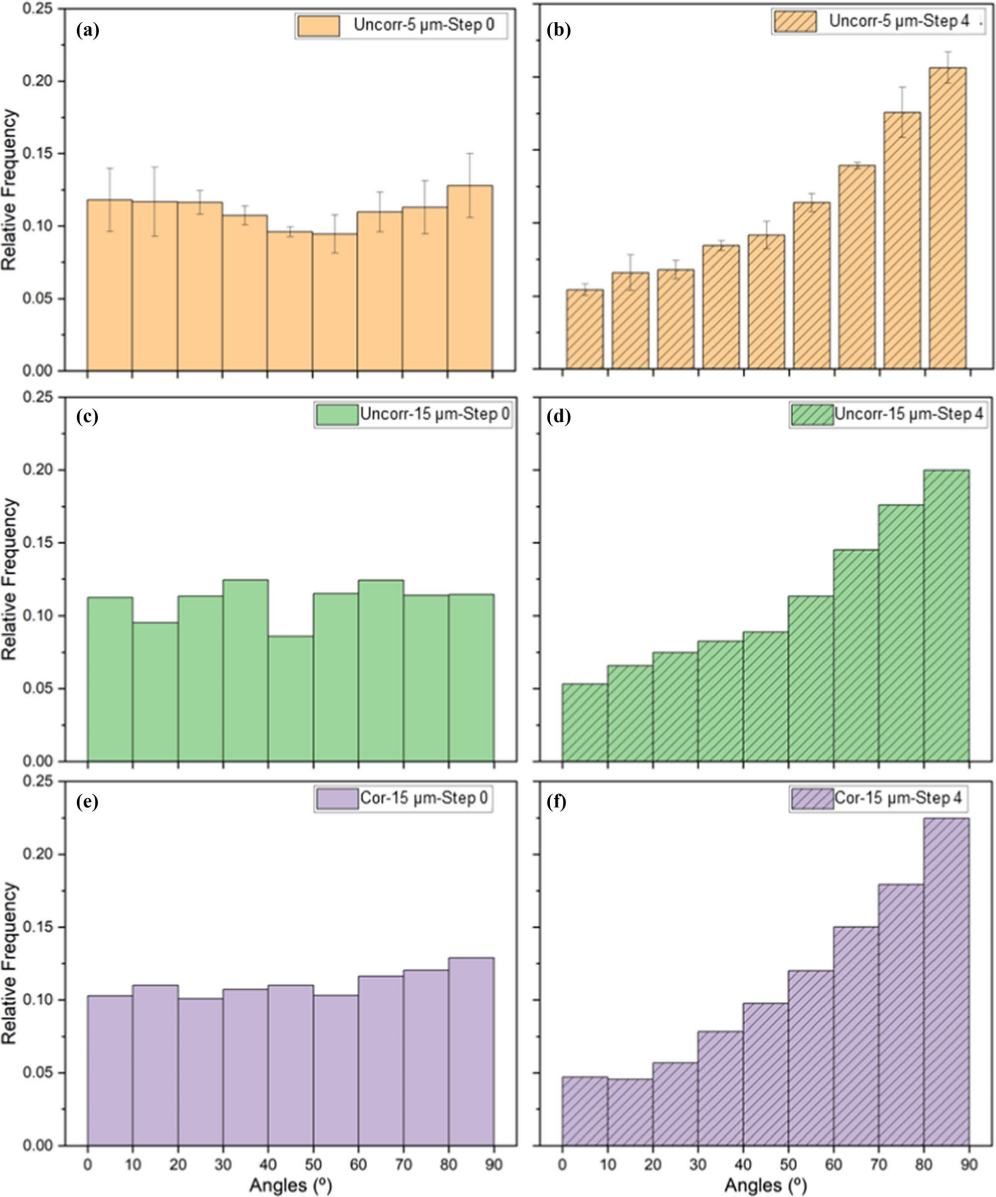
Histograms of crack orientation angle distributions for uncorroded 5 µm (**a**, **b**), uncorroded 15 µm (**c**, **d**), and corroded 15 µm (**e**, **f**) coated AZ31 Mg alloy samples, before (Step 0) and after (Step 4) deformation, respectively

Figure 6 presents histograms of crack orientation angles for uncorroded 5 µm (Fig. 6a, b), uncorroded 15 µm (Fig. 6c, d), and corroded 15 µm coated (Fig. 6e, f) samples, both before (step 0) and after deformation (step 4). In the initial, unloaded condition (step 0), all groups display a relatively broad and uniform distribution of crack orientations. After mechanical loading (step 4), each sample group exhibits a marked increase in the relative frequency of cracks oriented at 90°, corresponding to the direction perpendicular to the flexion axis. This shift is observed regardless of coating thickness or pre-corrosion.

### HR-DIC analysis of the in situ SEM images

**Figure 7:**
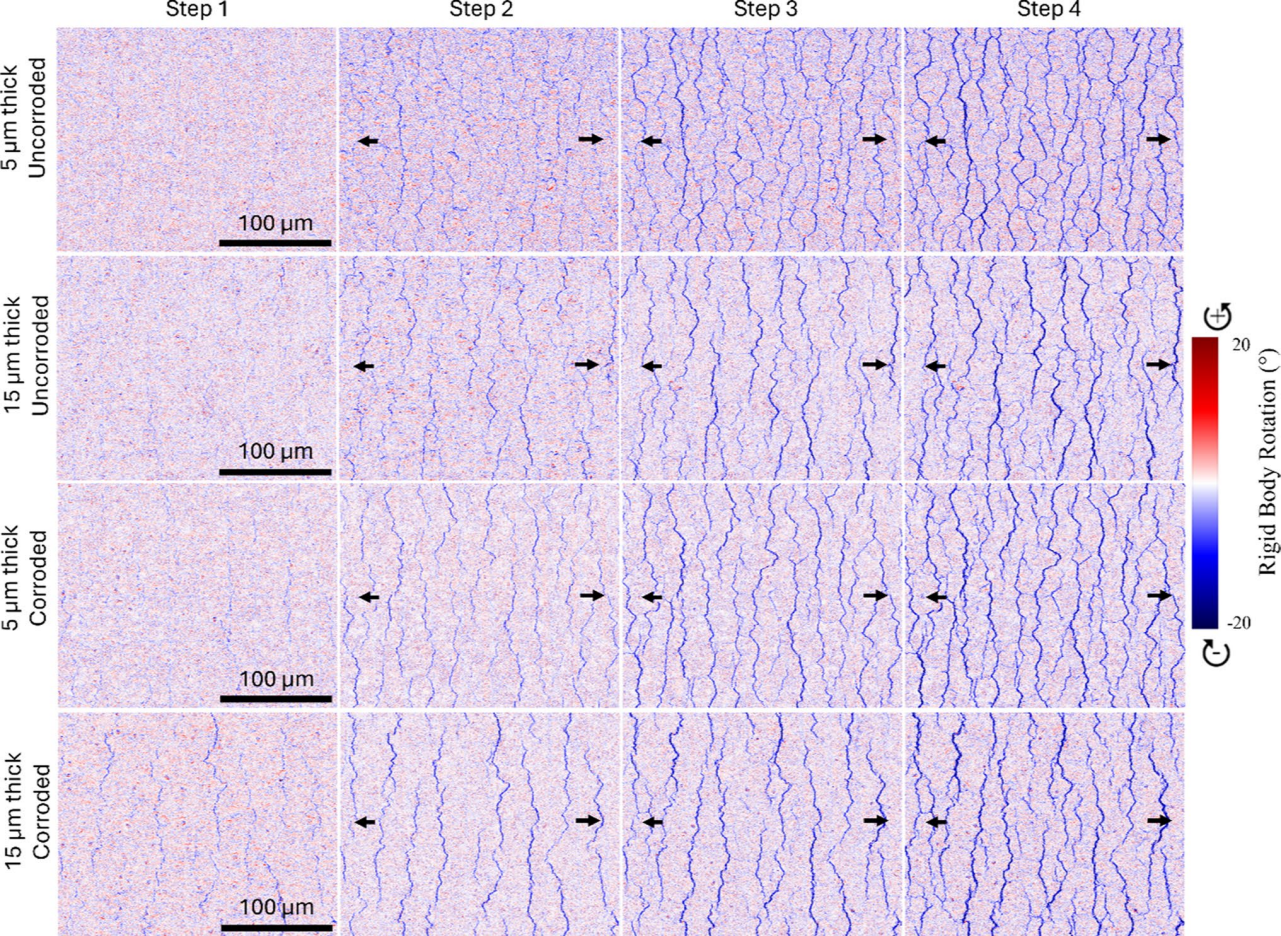
In situ DIC analysis results steps 1–4 (5 and 15 µm uncorroded and corroded coatings). Accumulated (total) $\text{RBR}$ maps displayed for step 1 (0.25 mm displacement), step 2 (0.5 mm displacement), step 3 (0.75 mm displacement), and step 4 (1 mm displacement) by using the SE images. The black arrows indicate the axis of flexion. The scale in the maps shows cracks in dark blue, while pink and red regions likely correspond to image noise and are not related to the strain

Quantitative analysis using RBR maps revealed notable differences in crack patterns between the 5 µm and 15 µm coated Mg samples (Fig. 7). The 5 µm coated samples exhibited a greater number of cracks. Generally, as strain increased, the number of cracks increased, and existing cracks became wider. In situ HR-DIC analysis of 5 µm and 15 µm coated samples via RBR maps (Fig. 7) revealed localized crack orientation patterns that intensified as the deformation increased. Initially, at low deformation (step 1–step 2), the active cracks were oriented orthogonal to the tensile axis. However, as strain increased (steps 3 and 4), cracks that initially formed orthogonal to the flexion axis deviated from this alignment, propagating in less orthogonal directions. This behaviour was more evident in the thinner coating. In contrast, the thicker coating exhibited less homogeneous strain distribution, characterized by islands of low strain separated by pronounced lines of higher strain.

Further analysis was conducted on corroded 5 µm and 15 µm coated Mg samples to examine the influence of corrosion on crack formation and propagation patterns (Fig. 7). The corroded 5 µm coated samples showed a higher number of cracks, similar to the non-corroded samples. However, the corroded 15 µm coated samples did not exhibit any significant increase in crack width. This implies that 5-day corrosion does not affect crack formation in either the thinner or thicker coatings.

**Figure 8:**
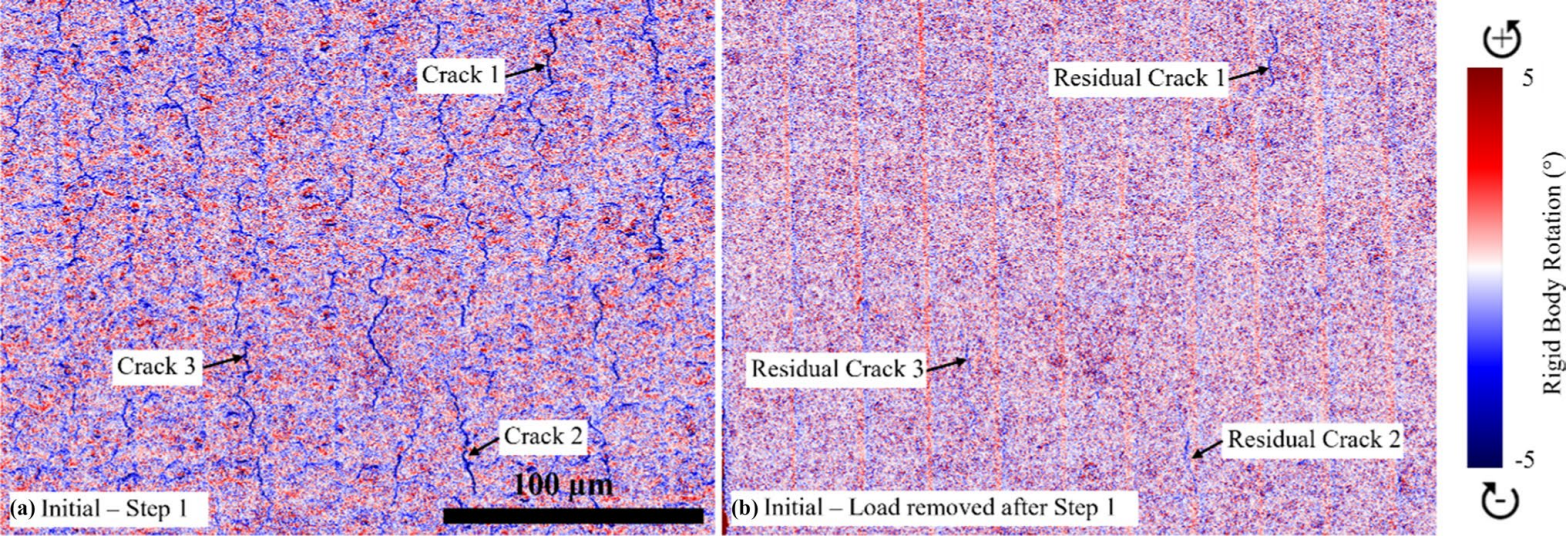
RBR map of the uncorroded 15 µm coated Mg sample at step 1 (**a**) and after load removal (**b**). Note that the vertical and horizontal noise in the RBR maps, particularly where individual photos have been stitched together, is unrelated to the surface strain and is a result of the stitching process. This noise should not be interpreted as indicative of actual strain on the surface

It is important to understand if these cracks closed up after the removal of load as residual cracks, even after loading may enhance corrosion. Crack behaviour during elastic loading in step 1 and following load removal is shown in Fig. 8 for an uncorroded 15 µm coated Mg sample. In step 1 (Fig. 8a), the applied elastic load revealed numerous cracks, where continuous lines of strain were evident. However, although the majority of cracks closed up when the load was removed, and although not as wide, some residual cracks (exhibiting a reduced width) remained indicating that damage to the coating was permanent.

#### Analysis of crack patterns

Tracking active crack propagation through SEM images during deformation proved impractical through inspection alone. Consequently, the HR-DIC technique was employed to accurately monitor crack progression. The cracking process began with the propagation of existing cracks according to Fig. 7. With continued deformation, new cracks oriented orthogonally to the tensile axis formed in the coating due to the substrate's deformation. Horizontal cracks also emerged at Steps 2, 3, and 4.

The HR-DIC analysis highlighted significant differences in crack segmentation width between the two coatings. Despite the higher number of horizontal cracks in the 5 µm coated samples, the total elongation of the surface remained similar between the two coatings (Fig. 7-step 4). The deformation in the 15 µm coated samples was associated with thicker, fewer cracks, whereas the 5 µm coating formed more numerous, thinner cracks.

**Figure 9:**
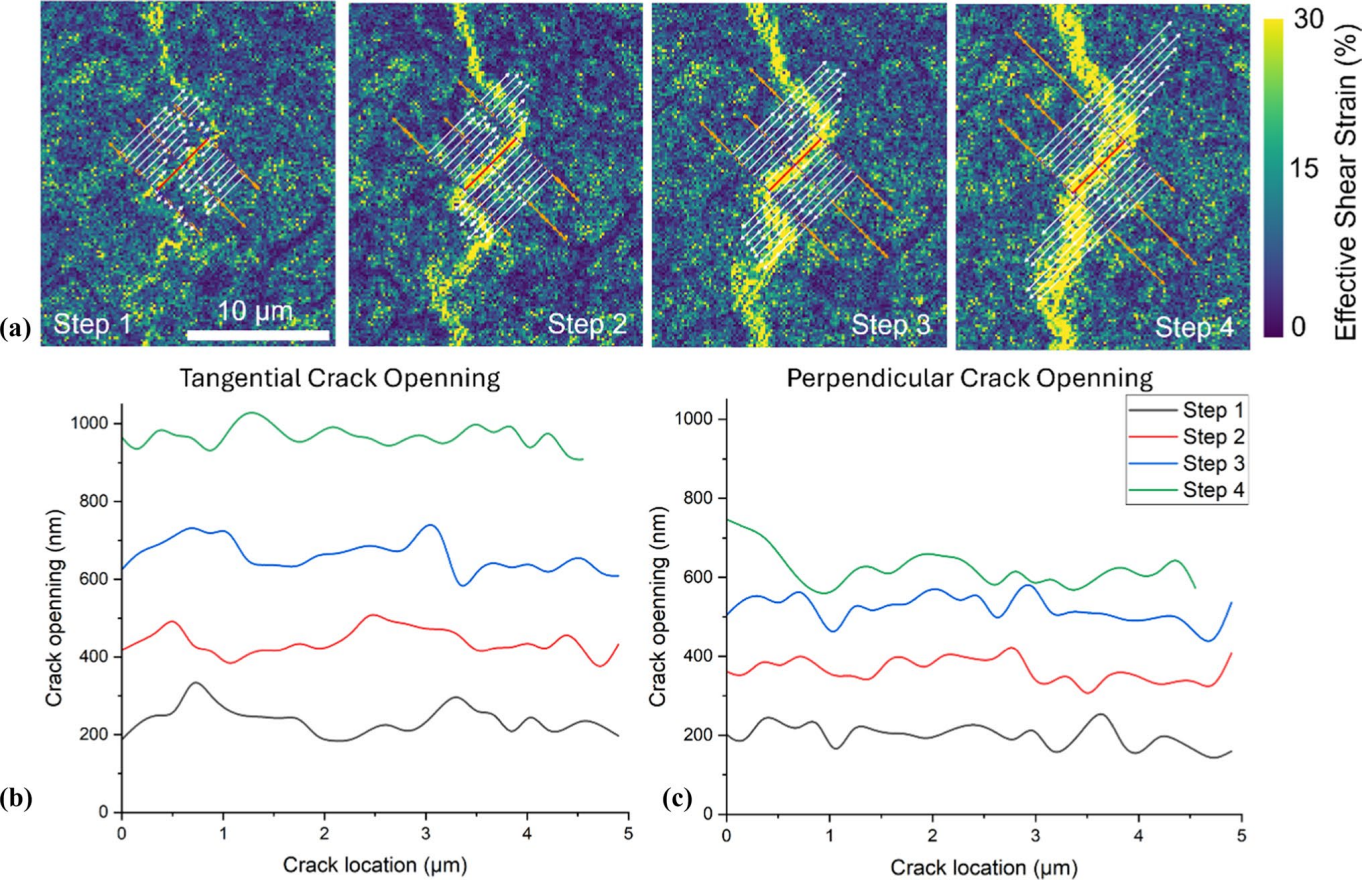
**a** ${\gamma }_{eff}$ map of a corroded 15 µm coated sample up to step 4, progressive crack propagation (Steps 1–4). Orthogonal measurement lines (7 pixels each side) indicate where local displacement vectors were analysed. **b**, **c** Incremental crack-opening profiles showing **b** tangential and **c** perpendicular displacements along the crack length. Although only three lines are visually presented for clarity, a total of 21 measurement lines were utilized to generate these graphs

In Fig. 9, a randomly selected crack approximately 14 µm in length and oriented at about 45° to the flexural axis was analysed in detail. The primary goal of selecting this particular crack was to clearly demonstrate, for the first time at high resolution, the incremental crack widening behaviour during deformation. The analysis, using the segmentation method described in Fig. 2, revealed that the crack-opening displacement is composed of both tangential and perpendicular components. Measurements were performed along segmented orthogonal lines crossing the crack line, enabling the extraction of incremental displacement vectors. Both tangential and perpendicular displacement magnitudes progressively increased with increasing applied displacement. By the final deformation step (Step 4), the tangential displacement reached approximately 966 ± 37 nm, while the perpendicular displacement reached approximately 623 ± 50 nm, confirming a bi-componential crack widening mechanism.

**Figure 10:**
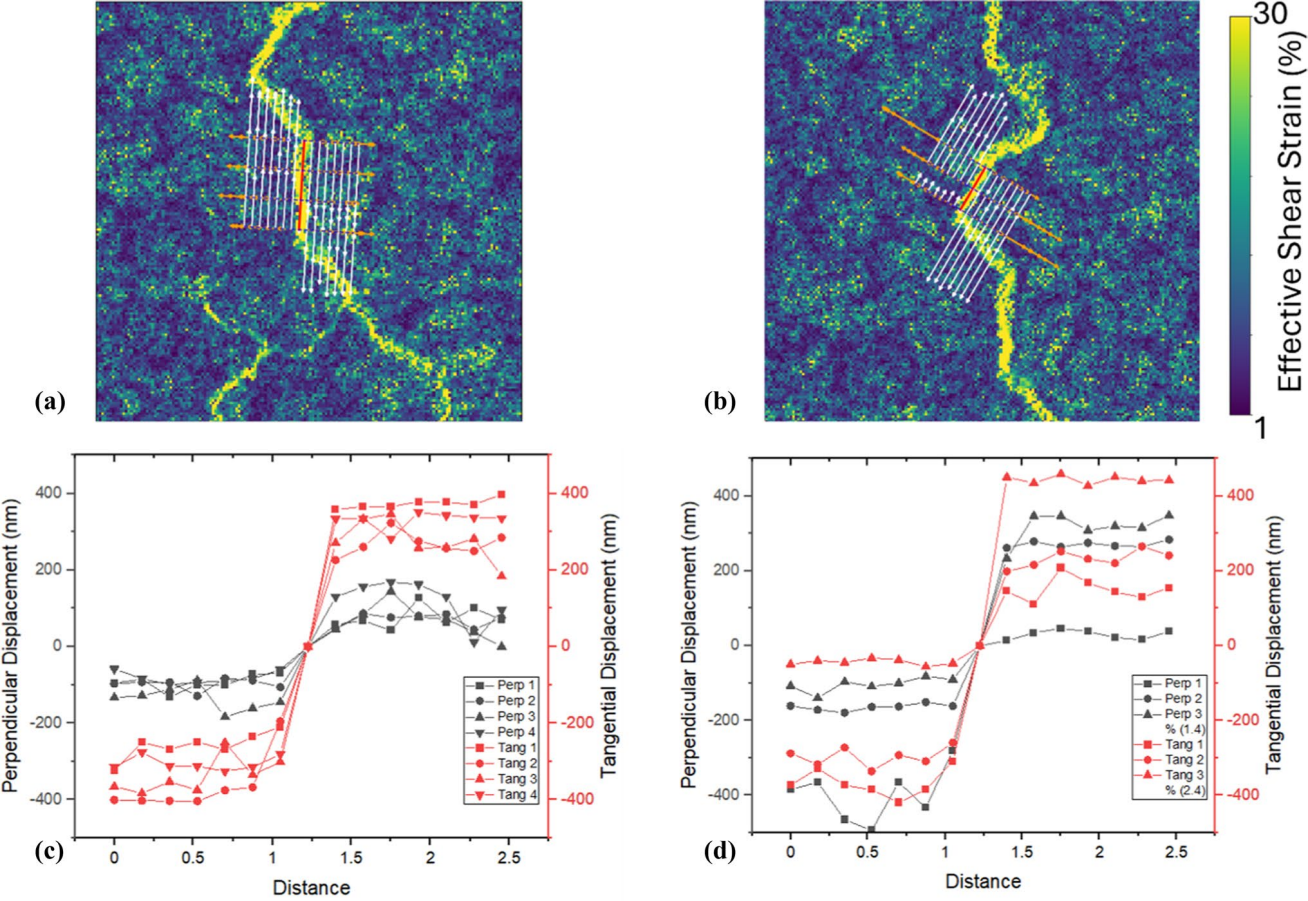
Comparison of crack displacement components at different orientations for a 15 µm coated Mg sample at Step 4. ${\gamma }_{\text{eff}}$ maps of a 15 µm coated sample at step 4 along 90° (**a**) and nearly 45° (**b**) cracks oriented to the axis of flexion and their **c**–**d** tangential and perpendicular

Additionally, Fig. 10 demonstrates similar displacement analysis for cracks oriented orthogonally (~ 90°) and again at ~ 45° to the loading axis. These additional analyses confirmed that crack widening consistently involved both tangential and perpendicular components, although tangential displacement generally dominated the crack-opening response.

#### Crack formation within the coating

**Figure 11:**
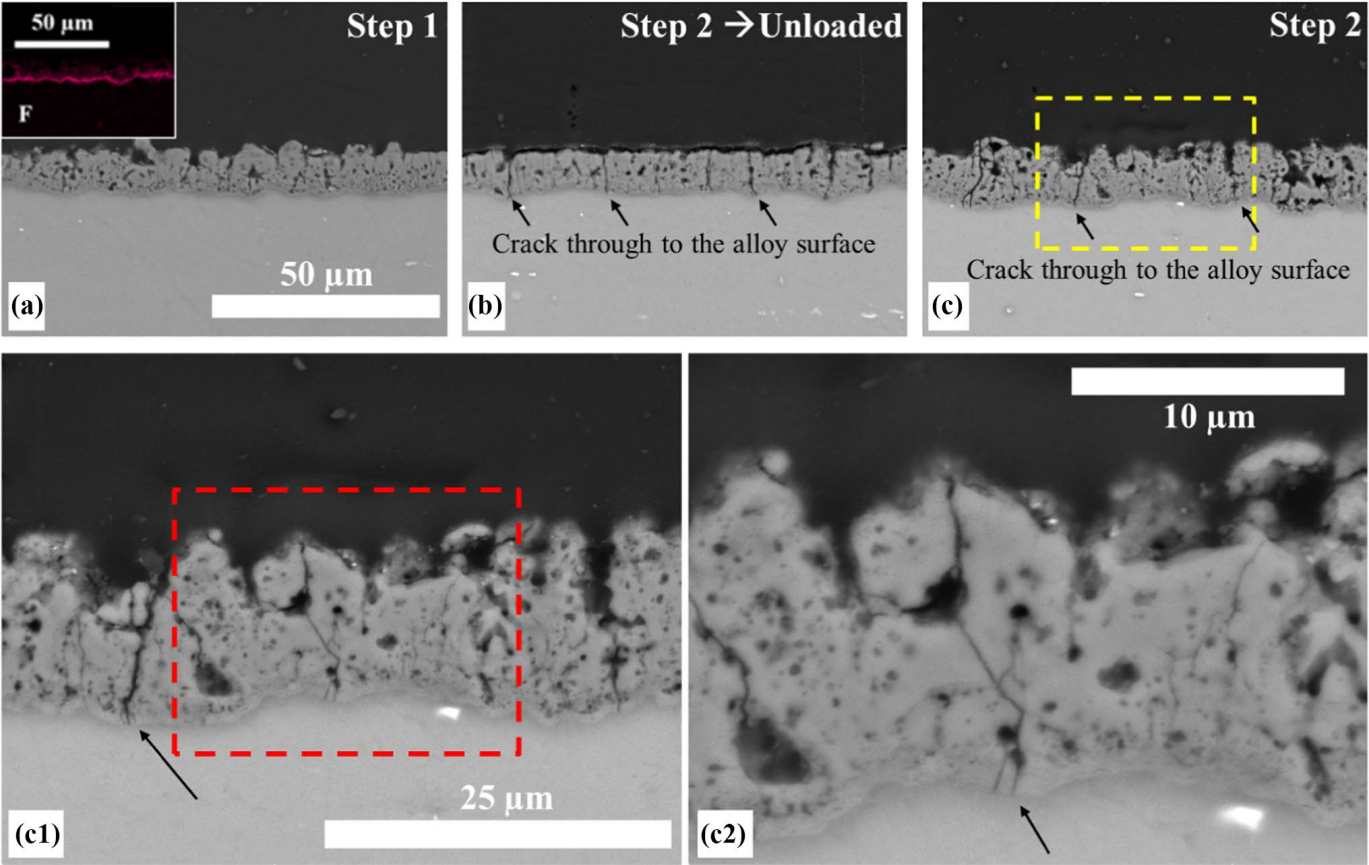
Cross-sectional SEM images of the 15 µm ECO-coated AZ31 Mg alloy at different loading steps: **a** Step 1 (unloaded), with inset showing the EDX map for fluoride highlighting the dense barrier layer at the coating/substrate interface; **b** after loading and unloading at Step 2, arrows indicate cracks propagating through the coating to the alloy surface; **c** loaded at Step 2, with yellow box indicating region of interest; **c**1 higher-magnification image of the boxed region in (**c**), and **c**2 further magnification, clearly showing through-thickness cracks reaching the alloy substrate

The DIC analysis showed that cracks formed on the surface of the coating when under tensile strain. It is important to understand if these cracks propagated through the coating to the alloy surface as this may affect corrosion. Comparisons were made between AZ31 rods with a 15 µm thick coating which were embedded while still under load (at step 4) and other bars which were loaded and unloaded before embedding and sectioning. SEM of the cross sections of the rods, specifically in the tensile region (Fig. 11), showed that deformation led to crack propagation through the coating and the dense fluorine-rich layer down to the alloy's surface. These cracks persisted even when the load was removed. Although these cracks may allow fluid to reach the alloy surface, there was no evidence of delamination or spalling between the coating and the substrate under either elastic or plastic load.

### Static loading of coated and uncoated AZ31 Mg alloy immersed in HBSS

**Figure 12:**
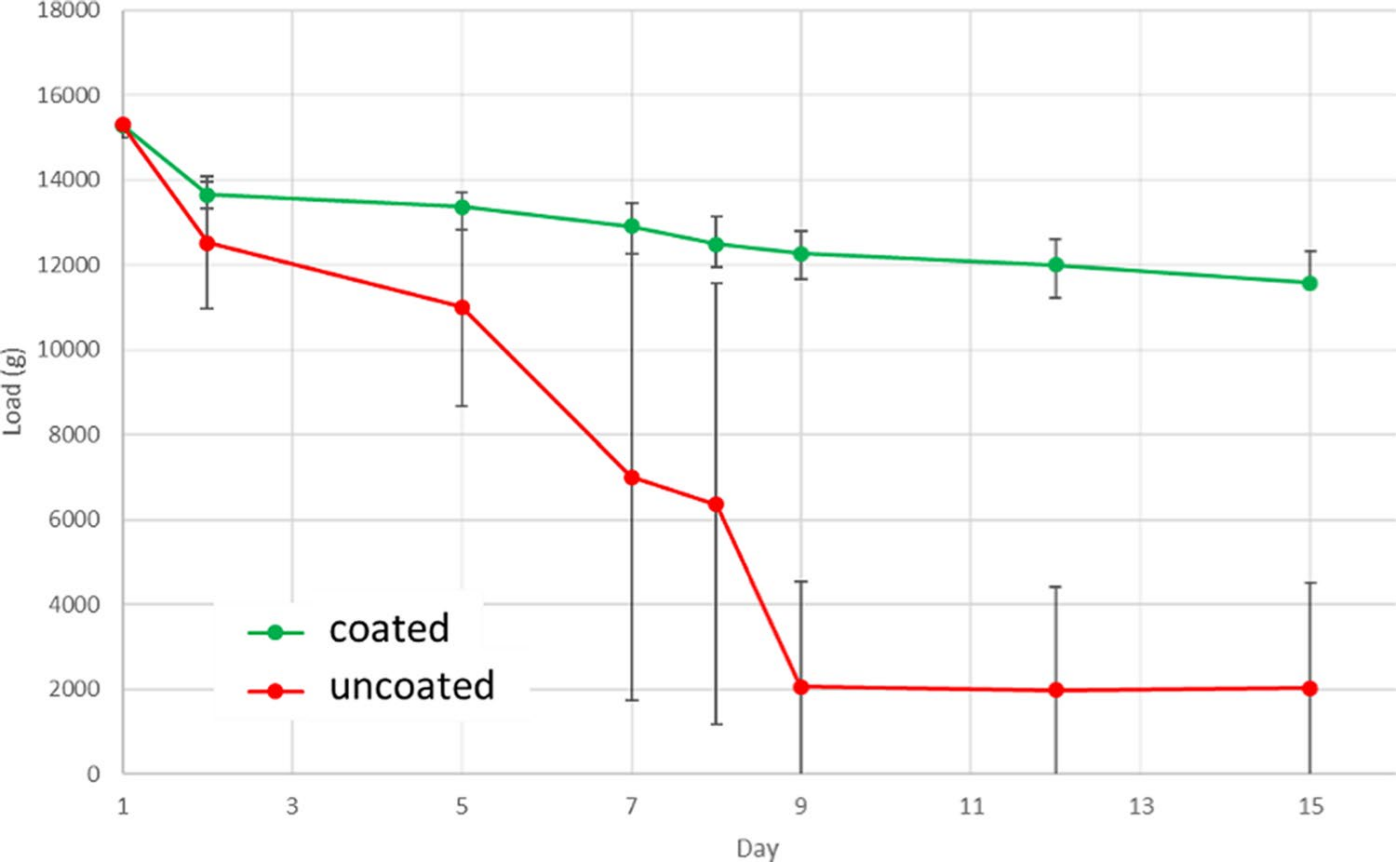
Load reduction over time for 3 mm bars of 15 µm coated (green line) and uncoated (red line) AZ31 Mg alloy immersed in HBSS under 3 PB test conditions

A static test was carried out in order to understand the effect of these cracks that formed in the coating due to high strain on the corrosion of the subsurface alloy. Figure 12 illustrates the reduction in load over time for 3 mm bars of 15 µm coated and uncoated AZ31 immersed in HBSS, initially loaded to 150 N in a 3 PB static test. The uncoated samples show a significant reduction in load, and on day 9, all the uncoated samples had mechanically failed. In contrast, the rate of decrease in load of the 15 µm coated sample was less and more linear, and all the samples remained intact when plastically deformed. Parametric assumptions were satisfied according to the Shapiro–Wilk test (*p* = 0.2290 for non-coated sample; *p* = 0.4973 for 15 µm coated). Multiple t-tests, corrected for multiple comparisons using Holm–Sidak, indicate significant differences between coated and uncoated samples after Day 9.

## Discussion

### PEO coating process and microstructural features

The form and content of the coating may be modified by the electrochemical deposition settings and by the compositional modifications of the electrolytes used. Depending on the electrolytes used, this approach can generate coatings comprising calcium and phosphate that are biocompatible, extremely adhesive, cohesive, and bioactive [40]. The PEO process, which is suitable for coating complicated 3D surfaces, uses an electrolyte bath of a proprietary dilute aqueous solution.

### Corrosion resistance and coating degradation

Implant degradation is the *raison d'être* for magnesium alloys, and controlled dissolution is required for biomedical viability. In this study, we have shown that ECO coatings reduce corrosion of the underlying magnesium alloy substrate by limiting exposure to the corrosive environment. The SEM analysis of unloaded samples revealed that the 5 µm coating exhibited a relatively smooth and uniform surface (Fig. 3), while the 15 µm coating exhibited greater roughness due to larger pores and more pronounced surface features, likely resulting from increased deposition time and layer buildup [41]. The cross-sectional analysis revealed that the multilayer structure of the coatings exhibits a dense, fluoride-rich barrier layer at the substrate interface, approximately 1 µm thick. After immersion in HBSS, the pores became wider, indicating coating degradation however, even as surface pores and cracks extended into the coating, the fluoride-rich barrier layer remained intact, and cracks did not reach the substrate indicating that this fluoride interlayer can mitigate rapid corrosion (Fig. 11).

The corrosion rates of uncoated and 15 µm coated AZ31 Mg samples in HBSS were measured over 14 days via weight loss measurement. Results showed negligible weight loss, making direct comparison between the coatings difficult. Notably, while corrosion-induced cracks and pores formed within the coating, the underlying barrier layer continued to provide effective protection. This is consistent with our previous findings [24], in which the 15 µm coated sample exhibited significantly lower corrosion rates than uncoated samples under more severe conditions (5 M NaCl), confirming the substantial protection provided by the ECO coating.

### In situ crack analysis by SEM and HR-DIC

To date, most research on the crack behaviour of coated samples has been conducted ex situ. However, for the first time, an in situ high-resolution three-point bending (3 PB) test was performed under a scanning electron microscope (SEM) on ECO-coated magnesium alloys at high resolution. HR-DIC was used to understand crack propagation through the coating under bending on the tensile region. The computational nature of HR-DIC mapping allows for the averaging of displacement fields over larger areas with high resolution (175 × 175 nm^2^ − 12 × 12 pixel^2^). This averaging process enhances the continuity of the crack network but also reveals a more complete picture of the material's structural integrity.

The differentiation between active and non-active cracks is critical for understanding the coatings’ integrity. Active cracks, which widen during stress, may compromise the protective nature of the coating by creating pathways for corrosive agents. This behaviour underscores the importance of monitoring crack propagation, as the expansion of these cracks under mechanical loading could lead to premature failure of the coating and subsequent exposure of the underlying Mg alloy to corrosive environments. Flexural stress applied during the 3 PB testing causes cracks to orient orthogonally to the flexion axis, aligning with the direction of maximum tensile stress.

Tracking the progression of active cracks through SEM alone proved impractical, as SEM captures both active and inactive cracks, some of which do not open or propagate under load and may be difficult to assess (Figure S2-c2 and c3). In contrast, the HR-DIC technique, specifically through RBR maps, enabled accurate monitoring of crack progression by highlighting localized changes. Although some noise is present in low-strain regions, RBR maps substantially improve the identification of propagating cracks, offering clearer insights into crack activity and orientation changes that develop with continued deformation. With this approach, it was observed that the cracking process began with the propagation of existing cracks, while new cracks, often oriented orthogonally to the tensile axis, emerged at higher deformation steps.

The crack behaviour differed notably between the 5 µm and 15 µm coatings. The 5 µm coating exhibited a higher density of cracks, initially and after deformation. This higher density may be attributed to the reduced thickness of the coating, which could lower its resistance to crack initiation and propagation. In contrast, the 15 µm coating showed fewer, but thicker, cracks, suggesting a different failure mechanism. The thicker coating may be able to sustain higher stresses before crack initiation, leading to fewer but more substantial cracks when they do occur.

Cracks develop on the coating surface in response to loading and with increasing strain propagate both through shear and tensile coating failure. Some of these cracks persist even when the load has been removed (Fig. 8). Under plastic loading (1 mm displacement) during the 3 PB tests, these cracks penetrate through the coating and the fluorine-rich barrier layer allowing the corrosive environment access to the alloy's surface exposing the alloy locally to the corrosive environment.

### Crack initiation and propagation mechanisms

Crack initiation and propagation in the coating were primarily governed by shear deformation rather than direct tensile opening (Figs. 9 and 10). This was evident even in regions where cracks were oriented perpendicular to the flexural axis, where tensile stress is typically maximal. The dominant role of shear likely stems from the combination of flexural loading and mismatched mechanical properties between the brittle coating and the ductile substrate. While the tensile surface of a bent specimen experiences axial tension, strain incompatibility at the coating–substrate interface introduces significant shear stresses. These shear components are critical in driving crack initiation, particularly at interfacial defects or weak zones. A recent study [31] supports this, showing that local plastic strain in AZ31 Mg alloy is highly heterogeneous, with values along slip bands and grain boundaries exceeding the average applied strain by an order of magnitude, further highlighting the role of tangential shear in coating failure mechanisms.

While the analysed crack in Fig. 9 was randomly selected rather than specifically chosen for representativeness, the primary intention of this detailed analysis was to provide a clear and quantitative demonstration of crack widening mechanisms using the methodology introduced in Fig. 2. This high-resolution displacement mapping successfully revealed that crack propagation under bending stress occurs through simultaneous contributions from tangential and perpendicular displacement components. The results strongly suggest that crack widening is incrementally sensitive to the applied deformation, regardless of the crack's precise orientation. We investigated the consequence of the angle of the crack on the displacements (Fig. 10) where the perpendicular displacement was less in the cracks that were orientated parallel to the tensile axis.

**Figure 13:**
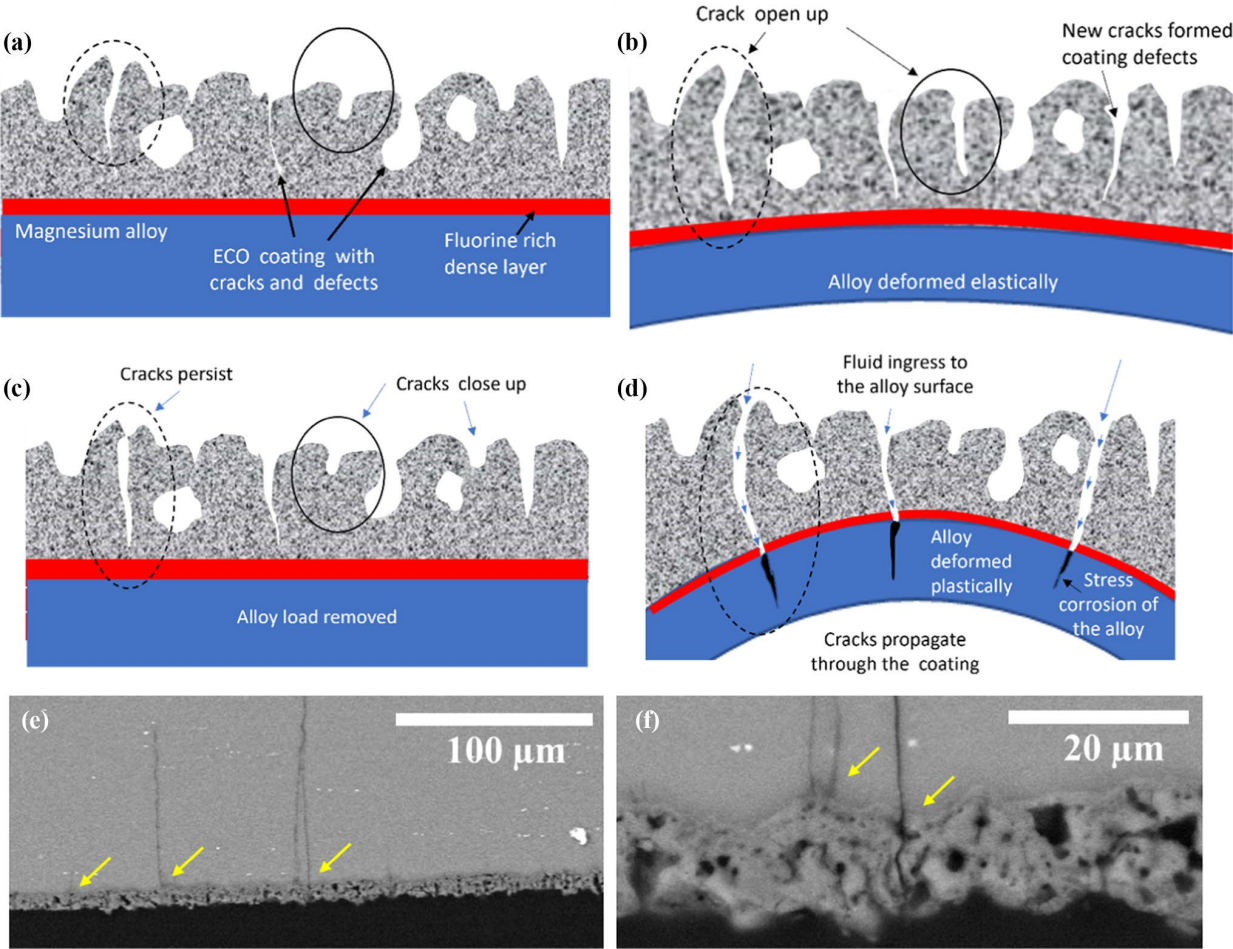
Schematic and microstructural evidence for crack evolution and coating failure in ECO-coated AZ31 Mg alloys under mechanical and corrosive loading. **a** Initial state: ECO coating exhibiting cracks and defects from fabrication. **b** Elastic deformation: Opening of surface cracks. **c** Load removal: Partial recovery and closure of some cracks, while others remain. **d** Plastic deformation: Propagation of cracks through the dense barrier layer to the alloy, initiating stress corrosion. **e**–**f** High-resolution SEM cross section after cyclic loading in HBSS, demonstrating a through-thickness crack connecting the external environment to the magnesium substrate (yellow arrows)

It is important to clearly differentiate the mechanisms of damage observed in ECO-coated AZ31 Mg alloys under mechanical and corrosive conditions. In this study, the majority of cracks detected within the coating are not the result of corrosion, but instead originate during the coating fabrication process and subsequently from mechanical deformation under loading. As schematically illustrated in Fig. 13(a–d), these pre-existing cracks and defects can open and widen in response to elastic or plastic deformation. Notably, while some cracks may partially or fully close upon unloading, others persist, leaving direct pathways for penetration of fluids from the surface to the underlying alloy.

Under increasing load, especially in the plastic regime, these cracks propagate through the entire thickness of the coating, including the dense, fluorine-rich barrier layer intended to protect the substrate. This through-thickness cracking fundamentally compromises the protective function of the coating. Once the coated material is exposed to a corrosive environment, these interconnected cracks act as preferred sites for localized electrolyte ingress, thereby accelerating corrosion processes and facilitating the onset of stress corrosion cracking in the underlying alloy.

To provide direct microstructural evidence for this mechanism, a high-resolution cross-sectional SEM images from coated samples following cyclic loading in HBSS (Fig. 13e–f) demonstrate cracks extending from the porous outer surface, traversing the entire coating, and penetrating into the magnesium alloy substrate. The crack path, highlighted by yellow arrows, visually confirms that deformation-induced cracks in the coating serve as conduits for environmental exposure of the alloy beneath. This observation supports the hypothesis that mechanical loading, particularly when combined with a corrosive environment, can drive crack propagation through the coating, fundamentally altering the degradation and failure behaviour of the system.

HR-DIC analysis of the surface combined with the cross sections through coatings and with the longer-term analysis of the effect of coatings in HBSS indicate that ECO coatings can protect magnesium alloys from corrosion even under loads that lead to permeant deformation. Although loading affects coating integrity and increases corrosion, uncoated magnesium alloys are more susceptible to loading. Previous tests that we have carried out on the same coatings on unloaded AZ31 incubated in HBSS resulted in very low corrosion after 2 weeks duration and only where the chloride level in the fluid was substantially increased was corrosion evident in coated AZ31 at this time [24]. Cracking in the alloy surface beneath the coating was not seen in the unloaded tests that we have previously carried out in high chloride levels. In this current study, cracks in the alloy were initiated from defects in the coating.

### Mechanical performance and clinical implications

Metallic fixation plates used for fracture stabilization in orthopaedic practice typically require intraoperative contouring for patient-specific anatomical fit [42], so magnesium alloys used in the clinic would likely be subjected to bending of a similar magnitude used in our study. The Young's modulus of AZ31 is usually around 45 GPa [43], which is more similar to the modulus of human bone (20 GPa) when compared to conventional metallic biomaterials such as titanium which has a modulus of 120 GPa. The modulus of ceramic coating is usually lower than that of magnesium alloys but often higher than the modulus of the surrounding tissues whilst ceramic coatings are also brittle. The differences in modulus between the implant and the coating combined with their brittle nature of the ceramic coatings may increase the stresses and failure in the coating particularly when the implant is subjected to bending. One ceramic material that is extensively used as a bioactive coating on orthopaedic implants is hydroxyapatite (HA). The Young's modulus of dense HA is 108 GPa [44] with flexural strength of 113 MPa [45]. However, due to poor adhesion with the underlying alloy spallation and delamination occur particularly for thicker coatings above 100 µm [46]. Gao et al. [47] investigated the static tensile and cyclic fatigue performance of PEO-coated samples and showed that the coating negatively affected the fatigue performance of the magnesium which was attributed to an increase in notching due to the PEO process. In their study, although surface cracks formed on the coatings, these cracks did not propagate through to the metal substrate as observed in our work. Instead, fatigue failure was primarily associated with cracks initiating at corrosion pits on the metal surface, likely due to loading remaining within the elastic regime. In contrast, the coatings examined in our study remained strongly adherent even during permanent deformation (beyond the yield point); however, such deformation led to the formation of through-thickness fissures, providing a pathway for corrosion.

Zheng et al. [48] working on a magnesium-zinc alloy, demonstrated that corrosion current density and weight loss after immersion in 0.9% sodium chloride solution increased significantly with applied tensile deformation, which was attributed to intergranular stress corrosion associated with high-density dislocations and deformation twins. There are both static and dynamic ASTM and ISO standards for investigating the corrosion behaviour of metallic alloys, and Kirkland et al. [49] provided a comprehensive review of recent methodologies for assessing corrosion in Mg-based alloys and biodegradable implant materials, outlining the advantages and limitations of both in vitro and in vivo methods. Unlike studies that focus solely on quantifying corrosion rates, using microstructural evidence our work demonstrates, using both schematic and microstructural evidence, how the integrity of the coating, and thus its protective capacity, can be affected under mechanical loading. We show that while the coating effectively restricts the access of the corrosive environment to the underlying alloy, the formation and propagation of cracks under load may compromise this barrier function. This underlines the necessity of assessing not only the corrosion rate, but also the mechanical durability and evolving protective performance of coatings under physiologically relevant loading conditions.

### Limitations and recommendations for future work

It is important to highlight some methodological and interpretative limitations of the current study. Our analysis employed a two-dimensional (2D) displacement field approach, inherently neglecting out-of-plane (*Z*-axis) material motion. The material deformation and crack propagation processes are three-dimensional (3D). Consequently, the current analysis does not account for potential *Z*-axis displacements, including vertical offsets of the surface topography, which could contribute to additional displacement components that were not captured in our analysis.

Furthermore, while crack-opening behaviours were examined for cracks oriented approximately at 45° and 90° to the flexural axis, boundary offsets and other microstructural heterogeneities at the surface were not quantitatively considered. These local offsets, particularly at crack boundaries, might play a significant role in crack propagation dynamics and displacement profiles.

Regarding the significance of the tangential displacement component, our analysis selected critical angles of 45° and 90° orientations. It should be noted, however, that cracks oriented parallel (0°) to the principal tensile axis were not analysed, as cracks in this orientation typically experience minimal opening under flexural loading conditions, resulting in negligible tangential or perpendicular displacement magnitudes. Therefore, such a scenario was considered beyond the scope of the current analysis due to its limited practical significance under the testing conditions employed.

The relationship between crack orientation and average crack-opening displacement (*δ*_avg_) analysis would enhance the mechanistic depth and general applicability of our results. While this angle-dependence relationship was not explored within the current study, it represents a valuable research direction for subsequent investigations. Future studies will utilize the developed displacement-analysis algorithm, along with cross-sectional speckle patterning methods, such as those demonstrated by Yavuzyegit et al. [31], to comprehensively explore both surface and subsurface displacement mechanisms and interfacial shear behaviour between the coating and AZ31 substrate.

Although microstructural coupling between crack trajectories and grain orientation is of great scientific interest,  Electron Backscatter Diffraction (EBSD) analysis of the coating–substrate interface posed considerable challenges in this study. The high thermal gradients inherent to the ECO process likely altered the substrate microstructure through localized recrystallization, complicating grain orientation analysis. Additionally, we analysed cracks in the coating surface, without propagation into the substrate, and therefore, did not observe any slip bands or intragranular deformation within the underlying alloy. Acquiring EBSD maps just beneath the coating would have required destructive sectioning or coating removal, which could interfere with crack path integrity. Future investigations using non-destructive techniques such as synchrotron X-ray diffraction or high-resolution TEM are recommended to overcome these limitations.

An additional limitation of the present study arises from the geometry of the tested samples. Rod-shaped specimens were employed, which inherently contributed to challenges during secondary electron (SE) imaging. Specifically, the curved geometry and incremental bending of the rod samples introduced noise and imaging artefacts, as certain surface regions intermittently moved closer to the electron gun during deformation. Consequently, consistent and uniform high-resolution SE imaging across the entire sample surface was difficult to maintain, leading to localized image quality variations.

Another limitation of the present study lies in the exclusive use of secondary electron (SE) imaging, which, while useful for highlighting surface topography, poses challenges for quantitatively interpreting deformation and crack behaviour. SE imaging is particularly sensitive to sample orientation and height variations—factors that became more pronounced due to the bending of rod-shaped specimens, leading to localized image distortion and contrast inconsistency.

In addition, we did not apply external speckle patterns onto the coating surface; instead, the inherent surface morphology of the coating was used as the characteristic pattern for DIC analysis. While this approach was effective to some extent, it also introduced limitations, especially for the thicker coatings, which exhibited rougher and more uneven surfaces. These morphological irregularities reduced the precision of displacement tracking. In future studies, applying a thin, uniform speckle pattern—particularly on thicker coatings—would improve measurement accuracy and allow for more reliable deformation analysis across different coating conditions. Complementary imaging techniques such as backscattered electron (BSE) imaging, combined with flat specimen geometries, are also recommended to reduce noise and enhance image clarity during in situ testing.

A key limitation of this study is the absence of a quantitative analysis of how corrosion influences interfacial stress redistribution between the coating and substrate. While the progression of cracks under mechanical and corrosive environments was characterized, understanding the precise evolution of stress fields at the interface would require advanced imaging and preparation techniques. To enable reliable cross-sectional digital image correlation, it would be necessary to modify the AZ31 substrate using gold remodelling methods. However, this would have introduced artefacts due to different strain patterns associated with the cross section.

These limitations and considerations underscore the importance of extending future analyses to full three-dimensional characterization of displacement fields and grain-boundary interactions. Future investigations should incorporate three-dimensional imaging methods and cross-sectional analyses to thoroughly understand out-of-plane effects and the influence of surface grain offsets on crack propagation and opening behaviour.

In addition to these experimental and analytical constraints, it is important to acknowledge that the current study was conducted entirely under controlled in vitro conditions and does not replicate the complex biomechanical environment present in vivo. Factors such as body fluid dynamics, biological tissue interaction, immune response, and dynamic loading from patient activity are not captured using in vitro testing. While the results provide valuable mechanistic insight, further investigations, including longer-term degradation and eventual in vivo studies, are essential to fully assess the clinical relevance and implant performance of coated AZ31 Mg alloys.

## Conclusion

This study investigated the microstructural characteristics and mechanical behaviour of electrochemical oxidation (ECO) coatings on AZ31 Mg alloys, with a focus on their performance in corroded and non-corroded conditions and under mechanical stress.

SEM analyses revealed distinct differences between 5 and 15 µm coatings, where the thicker coating exhibited rougher and more uneven surfaces with a higher initial surface porosity. Despite these differences, both coatings maintained a dense fluoride-rich barrier layer critical for corrosion resistance.

Cross-sectional analysis confirmed that while surface pores and cracks extended into the coating, they did not penetrate to the alloy surface, ensuring the protective barrier's integrity under various conditions. Corrosion tests in HBSS showed that the ECO coatings effectively mitigated corrosion, although prolonged exposure increased surface porosity, suggesting gradual degradation over time.

The in situ 3 PB test highlighted critical differences in crack initiation and propagation between the two coating thicknesses. HR-DIC analysis indicated that high shear strain regions are associated with crack initiation and propagation, emphasizing the importance of understanding crack behaviour under mechanical loading. As load increases, cracks continue to propagate and widen penetrating through the dense fluorine-rich layer to the alloy surface and are the sites for the initiation of stress corrosion cracking in the alloy (Fig. 13). This suggests that coatings used to reduce corrosion of magnesium alloys used for medical implants should be tested in a physiological fluid with realistic loads.

## Supplementary Information

Below is the link to the electronic supplementary material.

Supplementary file 1 (DOCX 7173 kb)

## Data Availability

Data will be made available on request.
