# Supplementary material for: High-resolution DIC analysis of in situ strain and crack propagation in coated AZ31 magnesium alloys under mechanical loading
Source: J Mater Sci. 2025 Aug 25;60(33):14708–30. doi: 10.1007/s10853-025-11243-4 (PMC12397167; doi:10.1007/s10853-025-11243-4)
Supplement: Supplementary file 1 — (DOCX 7173 kb) [file 10853_2025_11243_MOESM1_ESM.docx]

**Supplementary**


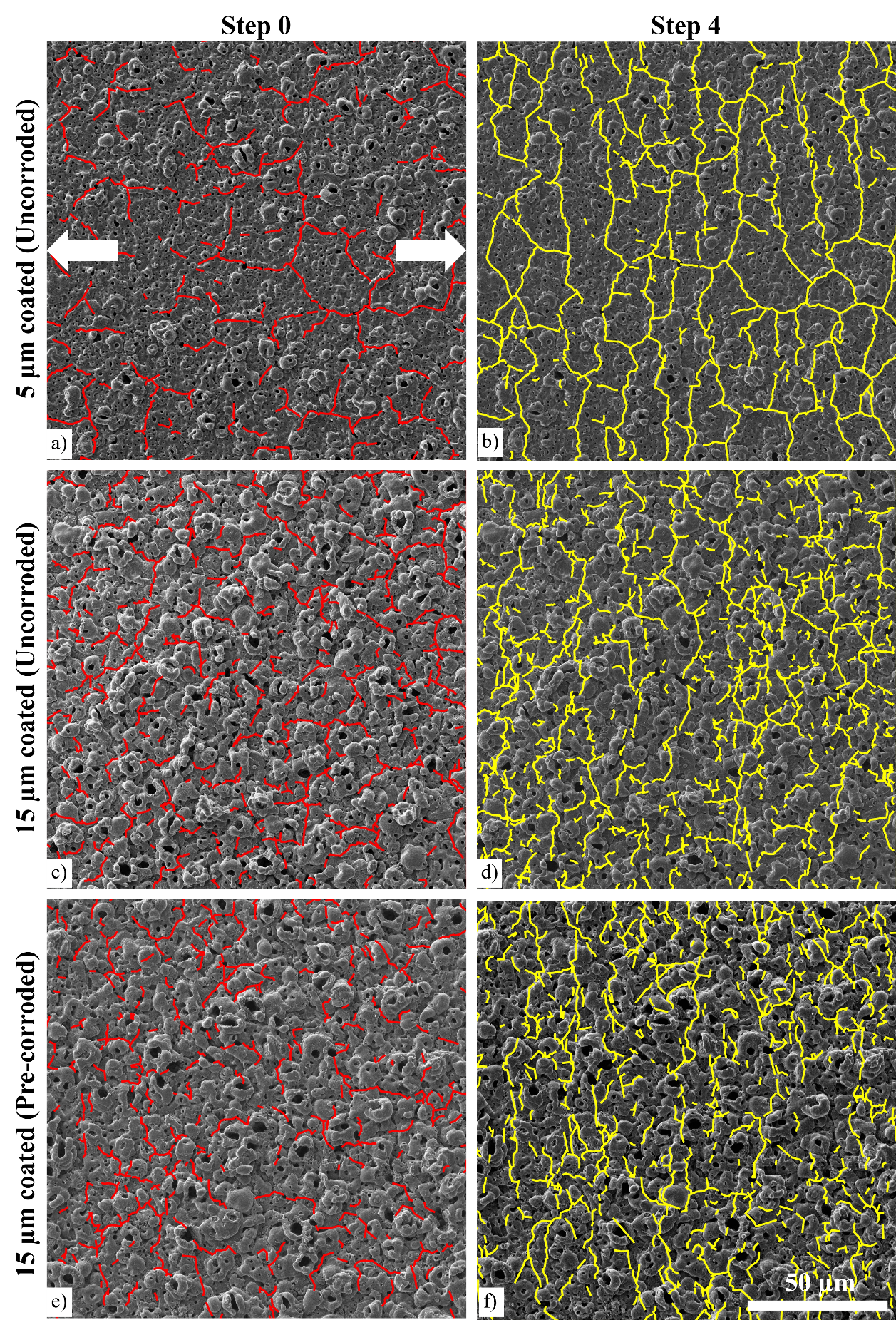


Figure S 1 Tracking crack orientation of 5 μm and 15 μm coated AZ31 Mg alloy on stitched SEM images: Step 0, non-deformed state (a-c-e), and final, deformed state at step 4 (b-d-f) of 5 μm and 15 μm. The tensile direction is shown on (a) as white arrow. Red and yellow lines highlight the locations of the cracks in the step 0 and final states, respectively.


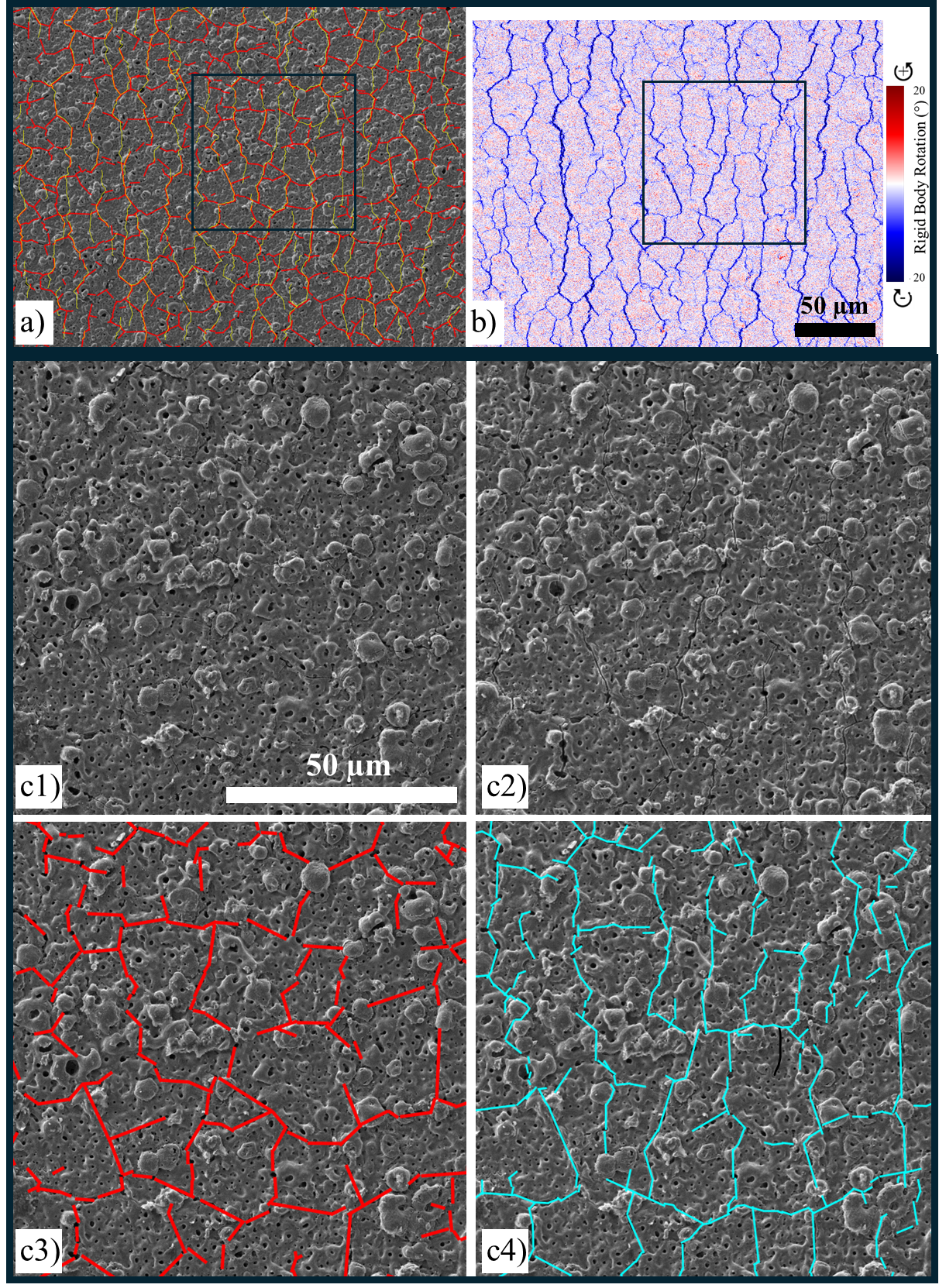


Figure S 2 Crack network identification and evolution analysis on the 15 µm coated/uncorroded AZ31 Mg alloy surface under in situ 3PB loading. (a) SEM image at step 0 overlaid with manually segmented cracks. Red lines represent cracks identified from the original step 0 image, while yellow lines indicate crack paths derived from tracking rigid body rotation (RBR) data between step 0 and step 4 using HR-DIC. (b) RBR map showing local rotational discontinuities associated with crack propagation; yellow crack lines in (a) were extracted based on this map. (c1–c4) High-magnification SEM images corresponding to the rectangular inset shown in (a) and (b). Images (c1) and (c2) represent the undeformed (step 0) and deformed (step 4, 1 mm displacement) conditions, respectively. Red lines in (c3) and cyan lines in (c4) mark the manually tracked cracks overlaid on the respective images, highlighting the evolution of the crack morphology under mechanical loading.
